# One-pot graphitic encapsulation of Fe_7_C_3_ nanoparticles enabling enhanced photothermal performance

**DOI:** 10.1039/d6nr01713d

**Published:** 2026-08-28

**Authors:** Chiara Puccinelli, Lorenzo Riccio, Erica Frostegård, Jana Dzíbelová, Christian Rentenberger, Jani Kotakoski, Jean-Marc Greneche, Belén Ballesteros, Cécilia Ménard-Moyon, Laura Maggini, Davide Bonifazi

**Affiliations:** a Institute of Organic Chemistry, University of Vienna Währinger Straße 38 Vienna 1090 Austria davide.bonifazi@univie.ac.at; b CNRS, Immunology, Immunopathology and Therapeutic Chemistry, UPR 3572, University of Strasbourg 67000 Strasbourg France; c Faculty of Physics, University of Vienna Boltzmanngasse 5 Vienna 1090 Austria; d Institut des Molécules et Matériaux (IMMM UMR CNRS 6238), Le Mans Université 72085 Le Mans Cedex 9 France; e Catalan Institute of Nanoscience and Nanotechnology (ICN2) CSIC and The Barcelona Institute of Science and Technology Campus UAB 08193 Bellaterra Barcelona Spain; f University of Strasbourg Institute of Advanced Study (USIAS) 67000 Strasbourg France

## Abstract

Here, we report a one-pot solvothermal synthesis that enables Fe_7_C_3_ nanoparticles (NPs) to direct the *in situ* formation of conformal graphitic carbon shells under comparatively mild conditions. Comprehensive structural analyses reveal that the resulting Fe_7_C_3_@C nanostructures consist of a crystalline carbide core encapsulated within a few-layer graphitic carbon shell, with small γ-Fe_2_O_3_ NPs by-products. Systematic variation of reaction parameters shows that both a reducing atmosphere and the Fe_7_C_3_ phase are essential for promoting graphitization, while Fe_5_C_2_ and Fe_3_C NPs prepared under analogous solvothermal conditions develop only amorphous carbon shells. This structural feature directly translates into superior photothermal behavior: Fe_7_C_3_@C NPs exhibit enhanced photothermal heating under 808 nm irradiation, even after 10 irradiation cycles, and a photothermal conversion efficiency (PCE) exceeding those of other iron carbides and several noble-metal nanostructures. These findings establish Fe_7_C_3_ as a uniquely capable phase for promoting *in situ* carbon ordering and highlight phase-dependent surface chemistry as a powerful tool for designing high-performance photothermal materials.

## Abstract 2

Fe_7_C_3_ nanoparticles drive *in situ* formation of graphitic carbon shells under mild conditions. This structural feature leads to superior photothermal performance surpassing those of other iron carbides and many noble-metal nanostructures.

## Graphical abstract

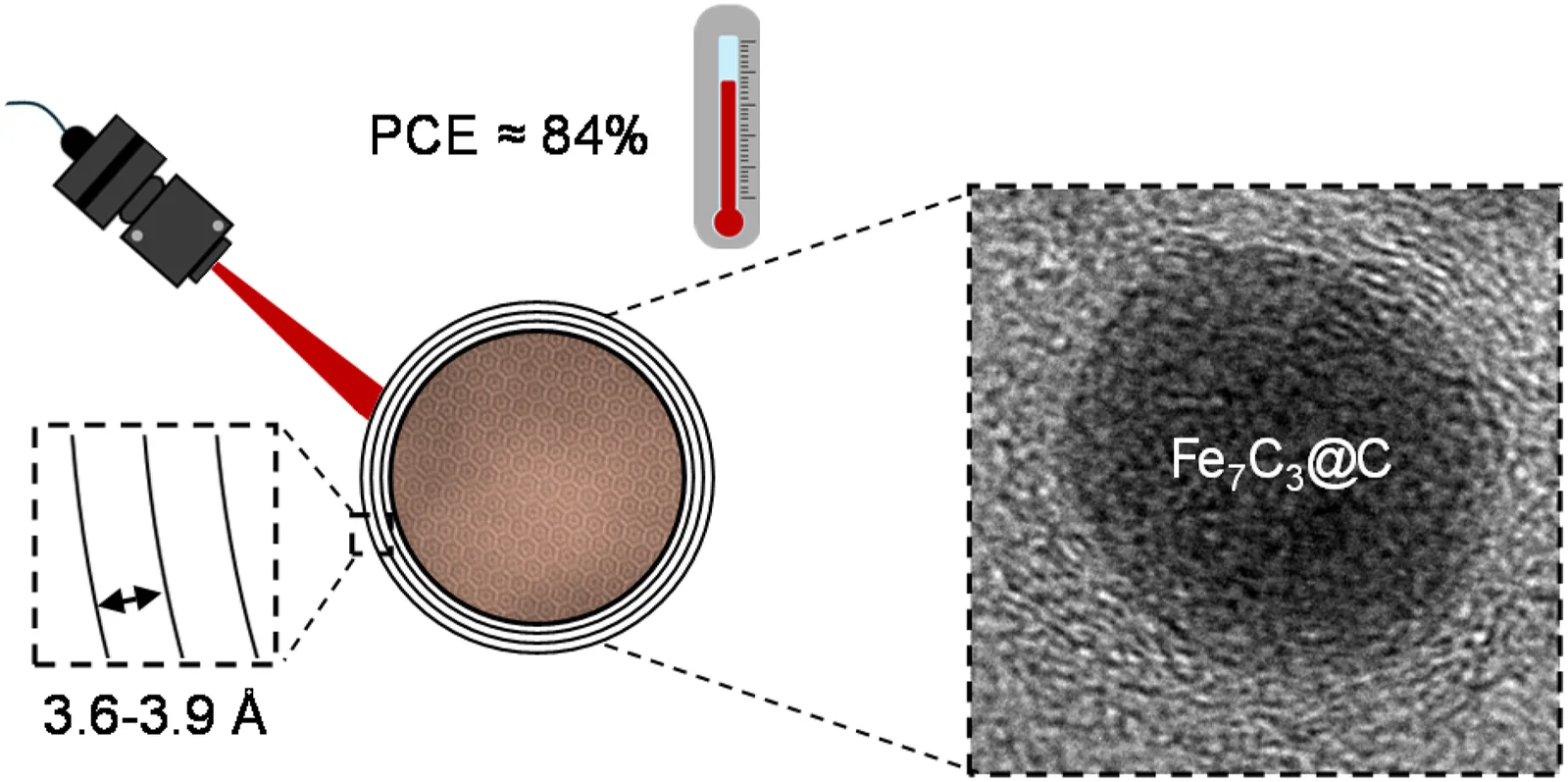

## 1. Introduction

Iron carbide NPs (Fe_*x*_C_*y*_) are intermetallic compounds in which carbon atoms occupy interstitial sites within close-packed iron lattices; the specific phase depends on the carbon coordination environment. Carbon atoms occupy trigonal–prismatic sites in Fe_3_C, Fe_5_C_2_, and Fe_7_C_3_, while octahedral sites are preferred in Fe_2.2_C and Fe_2_C.^1^ The insertion of carbon atoms not only strengthens the iron crystal lattice but also enhances its chemical inertness, making iron carbide both mechanically robust and chemically stable.^2^ Iron carbide nanoparticles have attracted significant interest for application in a broad spectrum of fields, primarily due to their magnetic properties (saturation magnetization approaching 140 emu g^−1^),^3^ ranging from magnetic data storage,^4^ bioimaging,^5^ catalysis,^6^ where their performances rival those of noble metals^7^ in critical industrial processes such as the Fischer–Tropsch synthesis,^2^ to magnetic and optical hyperthermia.^8^ Conventional preparation routes are divided into two main categories (Table 1): (i) Solid-state methods, which generally start from iron oxide nanopowders under various atmospheres (CO, N_2_, and Ar)^9,10^ or high-pressure/temperature synthesis (up to 8 GPa and 600–1600 °C).^11–13^ These approaches enable gram-scale synthesis; however, they typically produce particles with limited control over size, morphology, and phase purity. (ii) Solution-based methods, such as laser ablation^14^ or solvothermal routes,^2,15–19^ which operate under milder conditions (temperature below 350 °C and atmospheric pressure) and allow better control over the size and phase of the NPs.

**Table 1:** Synthetic routes for the preparation of iron carbide NPs of different phases

Synthetic route	Phase	Conditions	Ref.
Solid-state methods	Fe_3_C, Fe_7_C_3_	8 Gpa, 600–800 °C	Davydov *et al.*^11^
	Fe_3_C + Fe_1−*x*_C_*x*_@Carbon	8 Gpa, 1200–1600 °C	Baskakov *et al.*^12^
	Fe_7_C_3_ + Fe_1−*x*_C_*x*_@Carbon		
	Fe_3_C	N_2_, 800 °C	Schliehe *et al.*^10^
Solution-state methods	Fe–C	Laser ablation, Nd:YAG	Amendola *et al.*^14^
	Fe_2.2_C + Fe_5_C_2_	0.3 MPa, Ar and H_2_, 150 °C	Meffre *et al.*^17^
	Fe_5_C_2_	N_2_, 350 °C	Yang *et al.*^2^
		N_2_, 330 °C	Ge *et al.*^19^
	Fe_3_C	Ar + H_2_, 340 °C	Abel *et al.*^16^
		Ar, 310 °C	Yang *et al.*^18^
	Fe_7_C_3_	NH_3_, 350 °C	Zhao *et al.*^15^
	**Fe**_**7**_ **C**_**3**_ **@C (graphitic)**	**NH**_**3**_ **, 350 °C**	**This work**

Among the iron carbides, Fe_5_C_2_ NPs have the key feature of efficiently converting absorbed light into heat with a PCE, defined as the fraction of absorbed optical energy transformed into thermal energy, of 36.8%.^20^ This process, known as photothermia, is extremely relevant to cancer photothermal therapy,^21,22^ antibacterial treatment,^23^ and solar-driven catalysis.^24^ The growing interest in this class of materials is related to the necessity to find less expensive and sustainable alternatives compared to metallic NPs (particularly noble metals such as gold, which have long dominated this field)^25,26^ while maintaining broad optical absorption in the near-infrared (NIR) region and even enhancing PCE^20^ and photothermal stability.

During our investigation, we observed that Fe_7_C_3_, a phase that is comparatively underexplored despite its theoretically attractive electronic and magnetic properties,^27^ uniquely enables the *in situ* growth of conformal graphitic shells around the carbide core. Remarkably, this occurs in a one-pot synthesis at a relatively low temperature (350 °C). This is in contrast to other iron carbides, which are generally surrounded by an amorphous carbon shell, as also observed in our study (*e.g.*, Fe_5_C_2_ and Fe_3_C). The graphitic shell plays a crucial role in enhancing the stability of NPs by preventing physicochemical degradation^28,29^ and improving broadband light absorption,^30^ thermal conductivity,^31^ and biocompatibility.^32,33^

Specifically, we investigated the interplay between Fe_7_C_3_ synthesis parameters and the formation of the graphitic shell and showed that its presence directly translates into more efficient light-to-heat conversion compared to that with Fe_5_C_2_. Overall, these results provide new insights into the phase-dependent carbon-coating behaviour in iron carbide systems and establish Fe_7_C_3_@C as a promising platform for photothermal and catalytic applications.

## 2. Experimental

### 2.1. Materials

*N*,*N*-Dimethyloctadecan-1-amine (*N*,*N*-dimethylODA, 98%), octadecylamine (ODA, 90%), cetyltrimethylammonium bromide (CTAB, 98%), 1,2-hexadecanediol (98%), ammonium bromide (NH_4_Br, 99%) and oleylamine (70%) were purchased from BLD Pharm. Iron(0) pentacarbonyl (Fe(CO)_5_, 99%), iron acetylacetonate (Fe(acac)_3_, 97%) and cetyltrimethylammoniumchloride (CTAC, 98%) were purchased from Merck. Ammonium chloride (NH_4_Cl, 99%) was purchased from TCI. DSPE-PEG(2000)-NH_2_ (MW: 2000 Da) was purchased from Interchim. All reagents were used as received.

### 2.2. Synthesis of Fe_7_C_3_ NPs

A modified version of the procedure reported by Zhao *et al.*^15^ was used for the synthesis. 30 mL of *N*,*N*-dimethylODA were placed in a three-neck flask, stirred, and degassed under vacuum at 120 °C for 1 h. The atmosphere was replaced with NH_3_, and the mixture was heated to 180 °C. At this stage, 1.4 mL of Fe(CO)_5_ was injected under the NH_3_ atmosphere, and the mixture was maintained at this temperature for 30 min. During this process, the solution colour gradually changed from orange to black, indicating the decomposition of Fe(CO)_5_ and the nucleation of Fe nanostructures. The reaction mixture was heated to 350 °C at 5 °C min^−1^, held for 3 h, and then cooled to room temperature. The product was washed with EtOH and heptane and then collected using a magnet for characterisation.

### 2.3. Synthesis of Fe_5_C_2_ NPs

A modified version of the procedure reported by Yang *et al.*^2^ was used. 7.25 g of ODA and 0.113 g of CTAB were placed in a three-neck flask and degassed under vacuum for 30 minutes. The atmosphere was replaced with Ar, and the mixture was heated to 120 °C. At this stage, 0.5 ml of Fe(CO)_5_ was injected, and the mixture was heated to 180 °C at 5 °C min^−1^ and held for 10 minutes. During this process, the solution colour gradually changed from orange to black, indicating the decomposition of Fe(CO)_5_ and the nucleation of Fe nanostructures. The reaction mixture was heated to 350 °C at 5 °C min^−1^, held for 10 minutes, and then cooled to room temperature. The product was washed with EtOH and heptane and then collected using a magnet for characterization.

### 2.4. One-pot synthesis of Fe_3_C NPs

7.25 g of ODA, 0.113 g of CTAB, and 1 g of 1,2-hexadecanediol were placed in a three-neck flask and degassed under vacuum for 30 min. The atmosphere was replaced with Ar, and the mixture was heated to 120 °C. At this stage, 0.5 mL of Fe(CO)_5_ was injected, and the mixture was heated to 180 °C at 5 °C min^−1^ and held for 10 min. During this process, the solution colour gradually changed from orange to black, indicating the decomposition of Fe(CO)_5_ and the nucleation of Fe nanostructures. The reaction mixture was heated to 350 °C at 5 °C min^−1^, held for 10 min, and then cooled to room temperature. The product was washed with EtOH and heptane and finally collected using a magnet.

### 2.5 Two-step synthesis of Fe_3_C NPs

Fe_3_C NPs were prepared according to a literature procedure.^18^ 62.5 mmol of octadecene, 0.1 mmol of NH_4_Br and 1 mmol of oleylamine were stirred and degassed under a gentle Ar flow for 1 h in a three-neck flask. The solution was then heated to 100 °C and maintained at this temperature for 2 h before it was heated further to 180 °C to fully remove the organic impurities. After that, 5 mmol of Fe(CO)_5_ was injected into the reaction mixture and kept for 30 min. A colour change from orange to black was observed after *ca.* 1 min, indicating the decomposition of Fe(CO)_5_ and the formation of bcc-Fe NPs. To control the oxidation of the as-prepared bcc-Fe NPs, 2 mmol of oleic acid and hexane (0.5 mL) were added *via* a syringe and the resultant solution was aged at 140 °C for another 30 min before it was cooled down to room temperature. Acetone was added to precipitate the product. The product was collected using a magnet and re-dispersed in hexane. Fe_3_C NPs were then synthesized as follows: ODA (37.5 mmol), NH_4_Cl (0.12 mmol) and CTAC (0.19 mmol) were magnetically stirred in a three-neck flask and degassed under a gentle Ar flow for 1 h at 120 °C. Then, the resulting bcc-Fe NPs (5 mmol, in 10 mL hexane) were added *via* a syringe and the reaction solution was heated at 130 °C for another 30 min to remove hexane thoroughly before it was heated to the target temperature (350 °C) for 30 min. The black coloured solution was cooled down to room temperature by removing the heating source. Acetone was added to precipitate the product, which was collected using a magnet, washed with heptane : EtOH and re-dispersed in hexane.

### 2.6 Synthesis of γ-Fe_2_O_3_

Maghemite NPs were prepared according to a literature procedure^34^ by high temperature decomposition of iron organic precursors. The NPs were synthesized using iron acetylacetonate as the precursor and phenyl ether as the solvent. A mixture of 0.71 g of Fe(acac)_3_ (2 mmol), 2.38 g of 1,2-hexadecanediol (10 mmol), 1.69 g of oleic acid (6 mmol), 1.60 g of oleylamine (6 mmol), and 20 mL of phenyl ether was added to a three-neck flask. Then, the reaction mixture was heated under mechanical stirring and a flow of Ar gas until a temperature of 200 °C was reached. This temperature was maintained for 2 h, and then the solution was heated to reflux (254 °C) for 30 min under an Ar atmosphere. At the end, the solution was cooled down to room temperature. The NPs were obtained by precipitation with EtOH, collected with a magnet, and finally dried under argon flow.

### 2.7. Phospholipid surface modification

Surface modification of the NPs was performed according to a procedure^35^ in the literature. A DSPE-PEG(2000)-NH_2_ solution in CHCl_3_ (four times the weight of the NPs) was added dropwise over five minutes to the synthesized NPs in CHCl_3_ (1 mg mL^−1^) under sonication, and the mixture was then sonicated for an additional 30 min (SONOREX DIGIPLUS DL 512 H, 100% power). The solvent was completely evaporated under a gentle flow of Ar. Lastly, the dried product was dispersed in deionised water using sonication (final concentration: 1 mg mL^−1^).

### 2.8. High-resolution transmission electron microscopy (HR-TEM)

The synthesised NPs were characterised using an FEI Tecnai G2 F20 at an accelerating voltage of 200 kV or an FEI Titan 80-300 at an accelerating voltage of 200 kV. An aliquot of the NP suspension in EtOH was deposited onto a holey carbon grid (Ted Pella, Inc) or Au TEM grids covered with an amorphous carbon support foil (Quantifoil) and left to dry overnight.

### 2.9. Scanning transmission electron microscopy

High-angle annular dark-field scanning transmission electron microscopy (HAADF-STEM) images were acquired on an aberration-corrected Thermo Fisher Scientific Spectra 30-300 S/TEM operated at 200 kV. Atomic resolution images were acquired on an HAADF detector with a current of 50 pA and a beam convergence semiangle of 25 mrad. The analyses were performed on the same sample grids previously used for HR-TEM analysis to ensure consistency. STEM-EELS compositional maps were acquired using a direct electron detection Gatan Continuum K3 EELS spectrometer.

### 2.10. Powder X-ray diffraction

Information on the phase composition was obtained by powder X-ray diffraction (PXRD) using a D8 Advance (Bruker, Germany) diffractometer in Bragg–Brentano pseudo-focusing mode with a theta/theta geometry. Zero-background silicon single-crystal plates, coated with a thin layer of grease to fix the powders, were used as sample holders. For all measurements, the diffractometer was mounted with a one-dimensional silicon strip detector, LynxEye (Bruker, Germany). Patterns were determined in the 30° to 65° 2-theta range over 3 h with an acceleration voltage of 40 kV and a beam current of 40 mA.

### 2.11. Raman spectroscopy

Raman analysis was performed using a Renishaw inVia microRaman system equipped with a 532 nm laser. All spectra were recorded at a laser power of 0.18 mW, with three consecutive 25 s acquisitions. Three spots were analyzed, and the average of the three measurements was used to obtain the final spectrum. The samples were prepared by lyophilizing and placing the powder on copper tape.

### 2.12. Photothermal measurements

The photothermal capacity of the NPs was measured using a laser diode system from Roithner Lasertechnik (LOSBLD-0808-2W-C/P). The samples were diluted with Milli-Q water to the desired concentrations. Aliquots of 500 µL in 500 µL Eppendorf tubes were irradiated using an 808 nm laser (300 mW). A Flir One infrared camera was used to record the temperature.

### 2.13. Mössbauer spectrometry

^57^Fe transmission Mössbauer spectrometry was performed using a ^57^Co/Rh γ-ray source mounted on an electromagnetic drive using a triangular velocity form. The spectra were collected at 300 and 77 K using a bath cryostat. The sample consisted of a thin layer of powder containing approximately 5 mg of Fe cm^−2^. The Mössbauer spectra were first obtained at 300 K using a velocity range of −12 to +12 mm s^−1^ to verify the presence of typical magnetic sextets attributed to magnetically blocked Fe phases. They were then repeated over the velocity range, optimising hyperfine-structure resolution. The fitting procedure was carried out using the home-made unpublished MOSFIT software, involving magnetic sextets and quadrupolar doublets composed of Lorentzian lines. The isomer shift values are referenced to that of α-Fe at room temperature, while the source velocity was calibrated using α-Fe as a standard at room temperature. The hyperfine parameters obtained at 0 K are summarised in the study by Liu *et al.*^36^ Although the isomer shift and hyperfine field values are quite similar, the number of spectral components can be used to distinguish between the phases. A typical 77 K Mössbauer spectrum of Fe_7_C_3_ exhibits three magnetic components, similar to Fe_5_C_2_; however, Fe_5_C_2_ shows a significantly smaller hyperfine field. In contrast, Fe_3_C exhibits a single magnetic component, which must be fitted using two magnetic sextets.

## 3. Results and discussion

### 3.1. Synthesis and characterisation of Fe_7_C_3_@C NPs

Fe_7_C_3_ NPs were prepared by a solvothermal method using *N*,*N*-dimethylODA and Fe(CO)_5_ as carbon and iron precursors under an NH_3_ atmosphere. In a typical procedure, *N*,*N*-dimethylODA was first degassed to remove traces of oxygen, after which Fe(CO)_5_ was added under NH_3_ protection, and the mixture was maintained at 350 °C for 3 h. After the synthesis, the NPs were magnetically separated and purified (see the Materials section). The crystalline structure of the obtained magnetic material was analysed by powder PXRD (Fig. 1a). The pattern exhibits peaks characteristic of Fe_7_C_3_ (JCPDS-17-0333) and iron oxide (JCPDS 39-1346). Due to the broad reflections and the similar structure between magnetite and maghemite, the nature of the oxide phase could not be conclusively identified at this stage. Importantly, no additional reflections corresponding to secondary iron carbide phases were observed, confirming the phase purity of Fe_7_C_3_. To accurately assess the sample composition and identify the iron oxide phase, Mössbauer spectrometry was performed. At 300 K, the Mössbauer spectrum shows an asymmetric quadrupolar doublet with broadened lines (Fig. 1b). This spectrum can be well described by a superposition of quadrupolar components with different linewidths, isomer shifts, and quadrupolar splittings. Due to the limited resolution of the hyperfine structure, multiple models can be proposed, and it remains impossible to determine which fitting model provides the best physical solution. The refined hyperfine values are given in Table S1 (SI). Decreasing the temperature to 77 K reveals a highly complex hyperfine structure arising from multiple magnetic components. The final fitting model consists of three main components: (i) a magnetic component with broadened, asymmetric lines resulting from a discrete distribution of hyperfine fields with commonly fitted values of the isomer shift and the quadrupole shift, (ii) a quadrupolar component with broadened Lorentzian lines, and (iii) a second magnetic component comprising three magnetic sextets with Lorentzian lines, as illustrated in Fig. 1b. The refined values of the hyperfine parameters are listed in Table S1 (SI). The isomer shift of the first two components is clearly consistent with the presence of Fe^3+^ species, *i.e.*, ferric oxide phases, while that of the third component is attributed to iron carbide. In addition, the relative absorption areas of the oxide and metallic carbide phases are consistent with those observed at 300 K. The hyperfine structures observed at 300 and 77 K clearly indicate superparamagnetic relaxation. These results unambiguously suggest the presence of small iron carbide NPs surrounded by several layers of ferric oxides and very small ferric oxide NPs. At this stage, the presence of γ-Fe_2_O_3_ is evident, but it is difficult to discuss and identify the nature of the iron carbide phase and its different crystallographic iron sites, and to compare them with the microcrystalline analogous phase, due to the small size of the Fe_7_C_3_@C NPs, which causes structural distortion, and the proximity of the iron oxide, thus forming an interfacial atomic layer. In addition, the quadrupolar feature observed at 77 K is due to the presence of non-interacting small NPs of γ-Fe_2_O_3_. HR-TEM (Fig. 1c and S3, SI) confirms the presence of two distinct populations of NPs as revealed by the Mössbauer analysis: (i) larger core–shell NPs with an average size of 16 ± 4 nm (Fig. S12 and S13, SI) and (ii) smaller NPs with an average size of 5 ± 1 nm (Fig. S14, SI). A magnified HR-TEM image of the larger population of NPs clearly shows a dense iron-rich core (indicated by higher contrast) encapsulated by graphitic carbon layers (marked by white and orange rectangles) (Fig. 1d). The formation of a graphitic shell at a relatively low synthesis temperature was unexpected, as graphitization is typically achieved in a second step (*i.e.*, after NP synthesis) and requires a much higher temperature.^28,37^ HAADF-STEM characterization of an individual particle revealed the characteristic [100] crystalline face of hexagonal Fe_7_C_3_, with a visible line defect at the NP core (Fig. 1e). The measured spacing of the graphitic shell ranges from 0.36 nm to 0.39 nm (Fig. 1f), close to that of bulk graphite (0.335 nm).^38^ This difference can be attributed to the strain induced by the shells’ curved geometry and to imperfect stacking caused by lattice defects. Electron energy loss spectroscopy (EELS) analysis confirmed that the largest NPs consist of an iron carbide core surrounded by a graphitic shell, while the smallest NPs are composed of iron oxide (Fig. 1g). Finally, Raman spectroscopy (Fig. 1h) confirmed the presence of graphitic carbon in the sample, as evidenced by the characteristic G band around 1600 cm^−1^. The broad D band around 1330 cm^−1^ and the *I*_D_/*I*_G_ ratio of 0.79 (average of 3 samples) indicate a rather high degree of disorder in the sample.^39^ This suggests that the graphitic shell contains many defects that locally disturb the sp^2^ C arrangement.

**Fig. 1:**
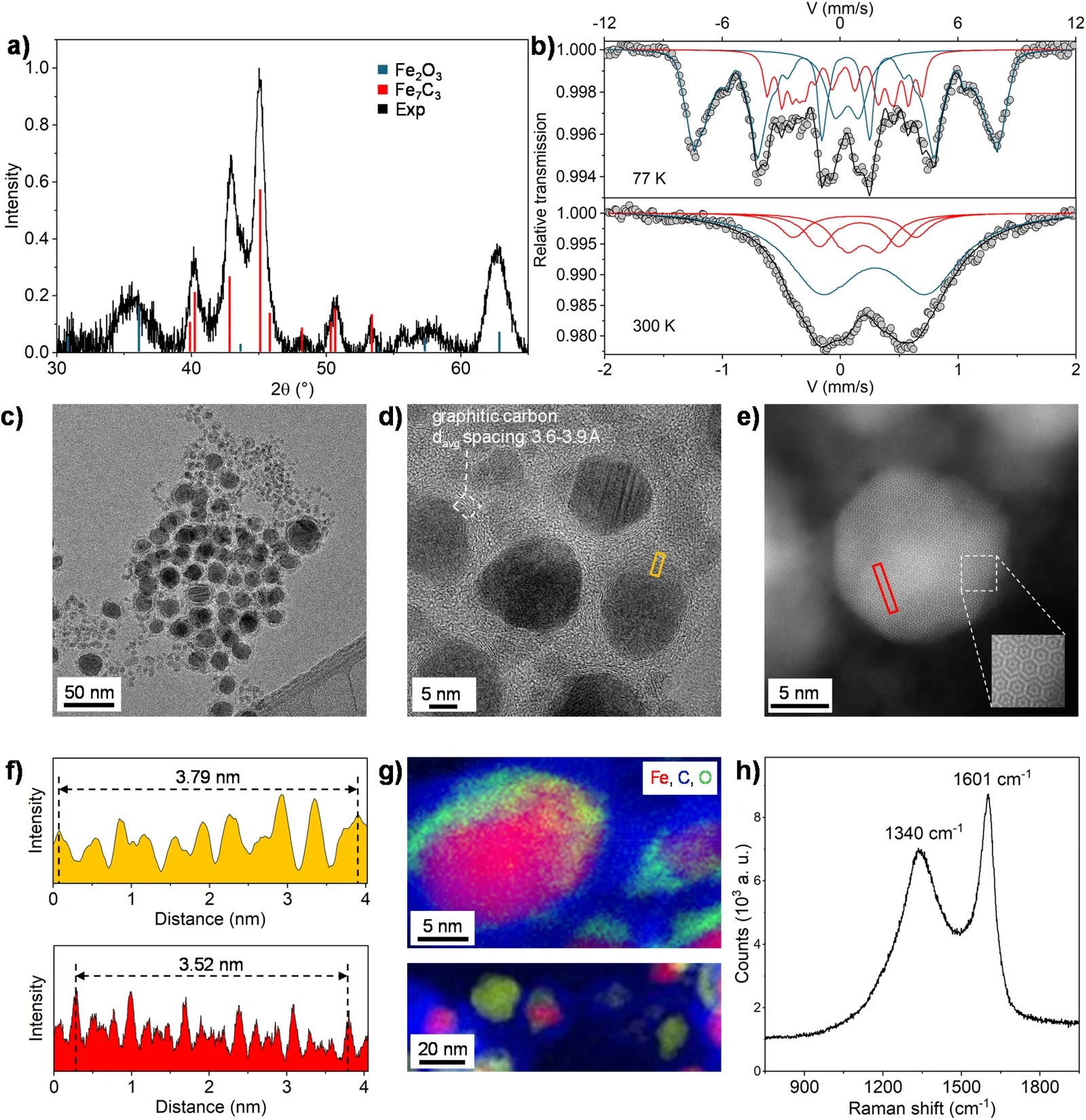
(a) PXRD pattern of Fe_7_C_3_@C NPs and the reference for Fe_7_C_3_ and γ-Fe_2_O_3_. (b) Mössbauer spectra of Fe_7_C_3_@C NPs at 300 and 77 K. Blue lines correspond to magnetic and paramagnetic contributions of γ-Fe_2_O_3_ while red lines correspond to magnetic and paramagnetic contributions of iron carbide. (c) TEM image of Fe_7_C_3_@C NPs. (d) HR-TEM image of Fe_7_C_3_@C NPs. (e) Atomic-resolution HAADF-STEM image of an individual Fe_7_C_3_@C NP. (f) Intensity profile recorded from the area indicated by the corresponding rectangular boxes in panels d and e. (g) EELS elemental mapping images of Fe_7_C_3_@C NPs. (h) Raman spectrum of Fe_7_C_3_@C NPs.

### 3.2. Experimental investigation of the factors governing graphitic shell formation

Given the unexpected formation of graphite-like outer layers on Fe_7_C_3_ NPs through a single-step, low-temperature one-pot process, we experimentally investigated parameters that could influence or enable graphite-like shell formation. All the reaction conditions tested are presented in Table 2.

**Table 2:** Overview of experimental conditions investigated to study the conditions affecting the formation of graphite-like carbon shells on Fe_7_C_3_ NPs

Entry	Fe(CO)_5_	*N*,*N*-dimethylODA	ODA	CTAB	Atm.	Phase
	Volume (mL)	Volume (mL)	Mass (g)	Mass (g)		
Std	1.4	30	—	—	NH_3_	Fe_7_C_3_@C
A	1.4	20	—	—	NH_3_	Fe_7_C_3_@C
B	1.4	40	—	—	NH_3_	Fe_7_C_3_@C
C	1.4	30	—	—	Ar	γ-Fe_2_O_3_
D	1.4	30	—	0.113	Ar	Fe_5_C_2_
E	1.4	—	14.5	—	NH_3_	γ-Fe_2_O_3_
F	1.4	—	14.5	0.113	NH_3_	Fe_5_C_2_
G	1.4	30	—	—	Ar : H_2_	Fe_7_C_3_@C
					95 : 5	

HR-TEM images show that the amount of *N*,*N*-dimethylODA neither affects nor alters the structural quality of the graphite, and it has no effect on the shell thickness (3.3 nm on average) (Fig. 2a and b). The influence of the reaction atmosphere was tested by performing the synthesis under Ar rather than NH_3_ (entry C). The PXRD pattern of the resulting magnetic material indicates the formation of iron oxide NPs (Fig. 2c).

**Fig. 2:**
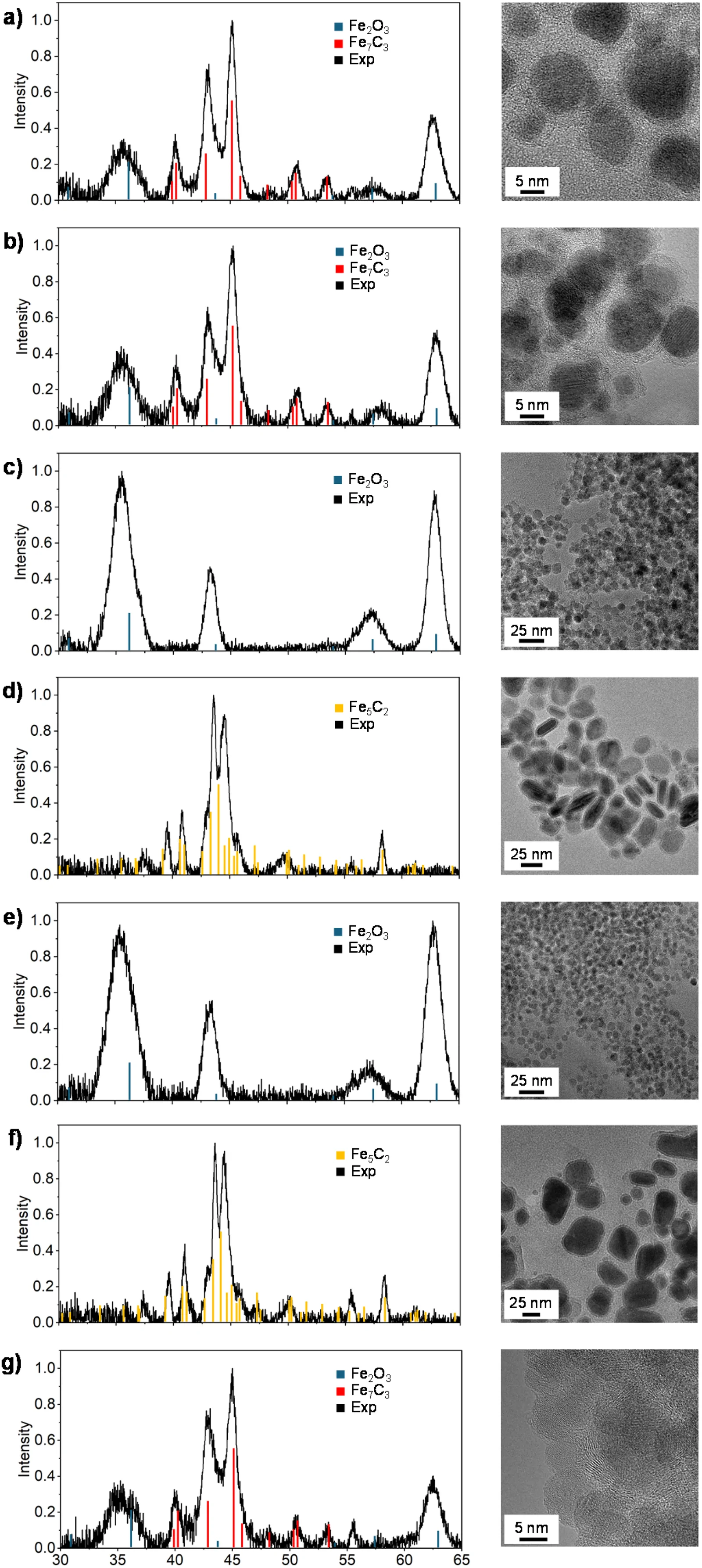
PXRD patterns and HR-TEM images of NPs obtained using different reaction conditions (panels a to g correspond to entries A to G in Table 2, respectively).

Consequently, it can be hypothesised that NH_3_ acts as an antioxidant during the formation of NPs. The previous experiment was replicated by adding a halogen source (CTAB), well known for its antioxidant role in the synthesis of Fe_5_C_2_ NP^2^, as an alternative to NH_3_ (entry D). The formation of iron oxide NPs was avoided, but Fe_5_C_2_ NPs with an amorphous carbon shell were obtained (Fig. 2d). Then, our standard protocol for Fe_7_C_3_ was performed, replacing the surfactant/carbon source with ODA, as it is commonly used for the synthesis of Fe_5_C_2_ NPs (entry E). In this case, as shown in the PXRD pattern, only iron oxide NPs were formed (Fig. 2e).

Entry E was repeated by adding CTAB to reproduce the standard synthesis of Fe_5_C_2_ under an ammonia atmosphere (entry F), resulting in the expected phase lacking a graphitic shell.

Finally, the synthesis of Fe_7_C_3_ NPs was performed by replacing NH_3_ with Ar : H_2_ 95 : 5 v/v % (entry G) to assess whether NH_3_ acts only as a reducing agent or plays a role in the formation of the graphitic shell. Some of the obtained NPs presented a graphitic shell around the carbide core. These results indicate that the primary role of NH_3_ is to act as a reducing agent. Under the synthesis conditions, NH_3_ dissociates into N_2_ and H_2_,^40^ with the latter maintaining a reducing atmosphere, avoiding the formation of oxidised species as with CTAB for Fe_5_C_2_.^41^ In conclusion, we experimentally observed that the formation of graphitic layers is phase-dependent and catalysed by iron in the Fe_7_C_3_ phase, which has previously been shown to exhibit higher catalytic activity than its Fe_5_C_2_ counterpart.^42–44^ This interpretation is further supported by the extensive literature demonstrating that iron-based nanomaterials can effectively catalyse the graphitisation of carbon and the formation of graphitic shells^37,45^ even if at higher temperatures than those reported in this study.

### 3.3. Synthesis and characterisation of Fe_5_C_2_ and Fe_3_C NPs

To elucidate the effect of the carbon shell structure (graphitic *versus* amorphous) on the photothermal behaviour of iron carbide NPs, we compared Fe_7_C_3_ with two other representative phases, Fe_5_C_2_ and Fe_3_C. Both phases were synthesised by a one-pot solvothermal method to ensure a meaningful comparison of their structural and photothermal characteristics. Fe_5_C_2_ NPs were obtained with a conventional solvothermal route using ODA as both a surfactant and a carbon source, CTAB as an inducing agent, and Fe(CO)_5_ as the iron source under an Ar atmosphere.^2^ Fe_3_C NPs were obtained using the same protocol in the presence of a long-chain diol such as 1,2-hexadecanediol^16^ (see the Materials section). The PXRD patterns exhibit characteristic reflections, respectively, of Fe_5_C_2_ (JCPDS 36-1248) and Fe_3_C (JCPDS 76-1877), thus confirming phase purity (Fig. 3a and b). The Mössbauer spectra of Fe_5_C_2_ and Fe_3_C samples obtained at 300 K and 77 K are shown in Fig. 3c and d. In the case of Fe_5_C_2_ NPs, the hyperfine structure at 300 K results from a magnetic component and a quadrupolar component, both characterized by broadened lines, whereas at 77 K, it results from the superimposition of one magnetic component with relatively narrow lines, described by means of three magnetic sextets, and a magnetic component with very broadened lines and a central quadrupolar doublet with broadened lines (Fig. 3c). It is important to note the use of two different velocity ranges to optimise the resolution of the respective hyperfine structures. The experimental spectra can be well described by various models using a discrete number of independent magnetic sextets and quadrupolar doublets. Among them, we report in Table S2 (SI) the refined hyperfine parameter values from a fitting model that simultaneously describes the spectra obtained at both temperatures. Two types of iron species can be easily distinguished based on their isomer shift values. Furthermore, the quadrupolar component observed at 300 K, which splits into a magnetic sextet and a weak quadrupolar component at 77 K, can be attributed to the presence of superparamagnetic relaxation phenomena of the iron oxides. The other magnetic components are consistent with those typically observed for Fe_5_C_2_, but the presence of other iron carbide phases cannot be formally excluded, given the similarity of the hyperfine parameters. At 300 K, both quadrupolar and magnetic components associated with Fe_5_C_2_ carbide were observed, which are in principle incompatible with the hyperfine structure expected for this crystalline phase. The paramagnetic contribution is likely related to the presence of a superparamagnetic phase. In fact, the size of the iron carbide particles estimated by TEM is about 30 ± 10 nm, while the size of the coherent domain is estimated at about 10–15 nm. This difference suggests that the particles consist of nanocrystalline grains separated by grain boundaries. As reported for various nanostructured systems,^46^ the structure of these boundaries is likely similar to that of a disordered FeC phase exhibiting paramagnetic behaviour. This explains the high complexity of the hyperfine structures observed in the Mössbauer spectra at both 300 K and 77 K. Furthermore, these findings support the conclusion that the carbide particles possess a nanostructured iron carbide core surrounded by an iron oxide shell. For Fe_3_C NPs, the Mössbauer spectra recorded at 300 K and 77 K (Fig. 3d) initially appear to show *a priori* only magnetic components (Fig. 3d). However, at 300 K, a quadrupolar doublet with broadened lines is necessary to well reproduce the experimental spectrum, particularly the central part. Based on the refined hyperfine data listed in Table S3 (SI), the largest isomer shift indicates that this quadrupolar component is due to Fe^3+^ species. In contrast, at 77 K, only magnetic sextets are required, and a magnetic sextet (outer lines) is observed, which is also characterised by the highest isomer shift, typical of the presence of Fe^3+^ species (Table S3, SI). Indeed, as previously described, the Fe_5_C_2_ and Fe_3_C NPs are composed of an iron carbide core and an iron oxide shell. TEM imaging (Fig. 3e, f, S4 and S5, SI) reveals particles with average sizes of 30 ± 10 nm (Fig. S15, SI) for the Fe_5_C_2_ NPs and 244 ± 97 nm (Fig. S16, SI) for the Fe_3_C NPs, while the sizes of the coherent diffraction domains are estimated at approximately 10 and 20 nm, respectively. Therefore, the nanostructured nature of Fe_3_C particles requiring at least 4 magnetic components with low hyperfine field contributions is consistent with the complex magnetic hyperfine structures observed at 300 K and 77 K, which differ significantly from those of crystalline Fe_3_C phases, due to the presence of grain boundaries. HR-TEM images show a core–shell structure, consistent with that in previous work^47^ (Fig. 3e and f). The particles consist of an iron carbide nanostructured core surrounded by a thin layer of iron oxide and amorphous carbon, in contrast to the graphitic layers observed in Fe_7_C_3_ NPs.

**Fig. 3:**
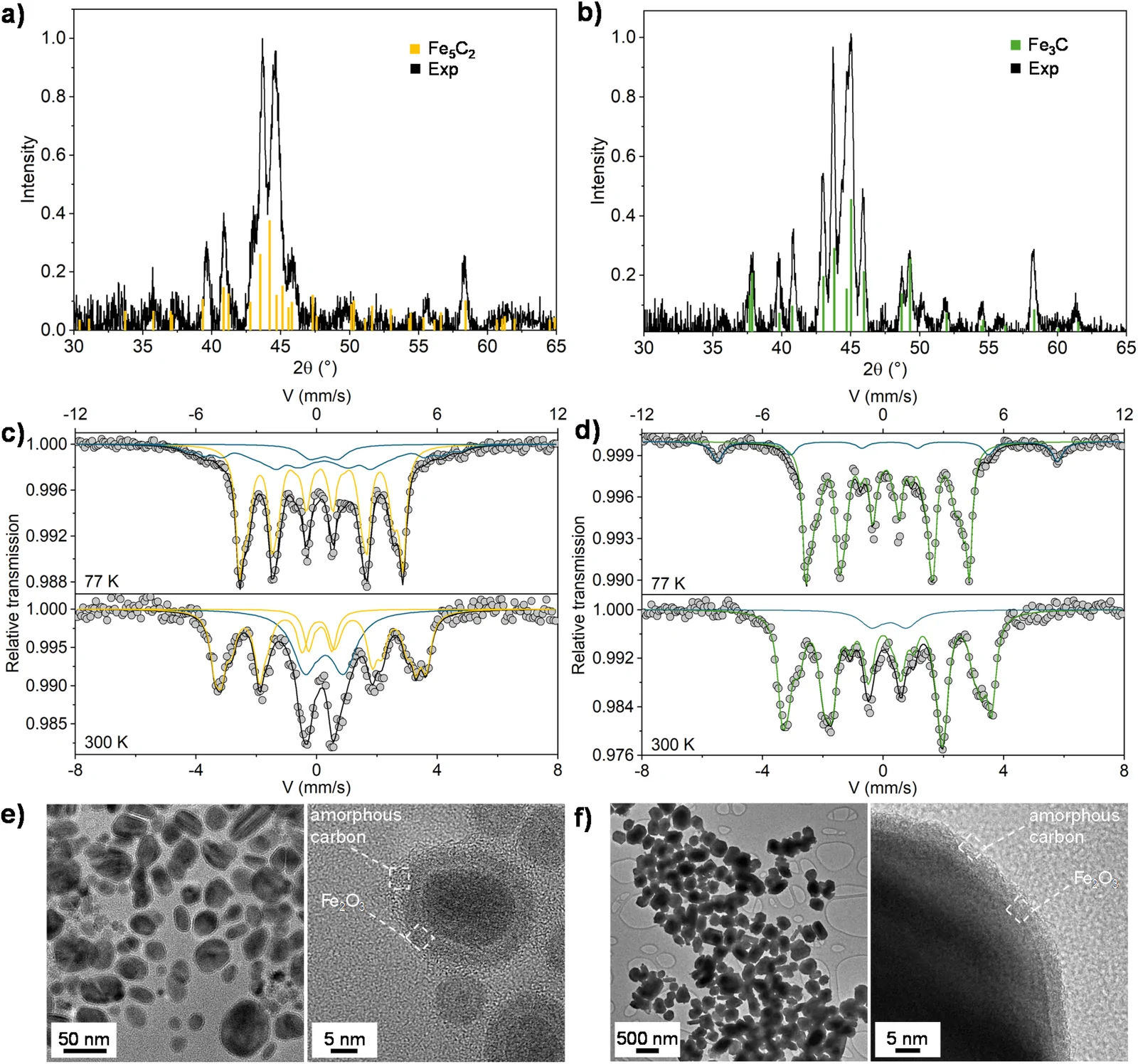
(a) PXRD pattern of Fe_5_C_2_ NPs and the reference for Fe_5_C_2_. (b) PXRD pattern of Fe_3_C NPs and the reference for Fe_3_C. (c) Mössbauer spectra of Fe_5_C_2_ NPs at 300 and 77 K. Blue lines correspond to magnetic and paramagnetic contributions attributed to external iron oxide layers, while yellow lines correspond to a core of iron carbide. (d) Mössbauer spectra of Fe_3_C NPs at 300 and 77 K. Blue lines correspond to magnetic and paramagnetic contributions attributed to external iron oxide layers, while green lines correspond to a core of iron carbide. (e) TEM and HR-TEM images of Fe_5_C_2_ NPs. (f) TEM and HR-TEM images of Fe_3_C NPs.

### 3.4. Photothermal properties of Fe_7_C_3_@C NPs

To evaluate the photothermal performance of iron carbide (magnetic characterization reported in Fig. S19–S21, SI) and assess whether the graphitic carbon shell of Fe_7_C_3_ NPs could enhance heat generation, the three phases and maghemite as a control (TEM images in Fig. S10 and an XRD diffractogram in Fig. S22, SI) were stabilized in water using a phospholipid-polyethylene glycol (PEG) derivative, which improves the colloidal stability and dispersibility of NPs in water.^35^ Following a protocol described previously (see the Materials section), 50 µg mL^−1^ aqueous suspensions of Fe_7_C_3_@C, Fe_5_C_2_, ∼244 nm Fe_3_C, ∼33 nm Fe_3_C (synthesized with a two-step protocol for meaningful size comparison, Fig. S17, SI) and ∼6 nm γ-Fe_2_O_3_ (as a control, Fig. S18, SI) NPs were prepared (TEM images in Fig. S6–S10 SI). The photothermal heat generation of the NPs was measured using a custom-built setup under continuous laser irradiation (808 nm, 0.5 W cm^−2^) for 10 min. As shown in Fig. 4a, Fe_7_C_3_@C exhibits a temperature increase (Δ*T*) of approximately 14 °C after 10 min irradiation, more than twice those of Fe_5_C_2_ (6 °C), ∼244 nm Fe_3_C (4 °C), ∼33 nm Fe_3_C (3.4 °C), and ∼6 nm γ-Fe_2_O_3_ (1.7 °C), while no significant temperature increase was observed in the control sample of water. The higher photothermal performance of Fe_7_C_3_@C NPs compared to that of the two other phases is likely due to the presence of the graphite-like shell. Furthermore, photothermal measurements were performed on three independently synthesized Fe_7_C_3_@C samples and they showed comparable heating behaviour despite differences in the graphitic shell's defect density, suggesting that, within the range of defect density obtained in this study, the photothermal response is not significantly influenced. Notably, this heat generation was achieved using a low NP concentration, highlighting their potential applicability in biomedical contexts beyond catalysis.

**Fig. 4:**
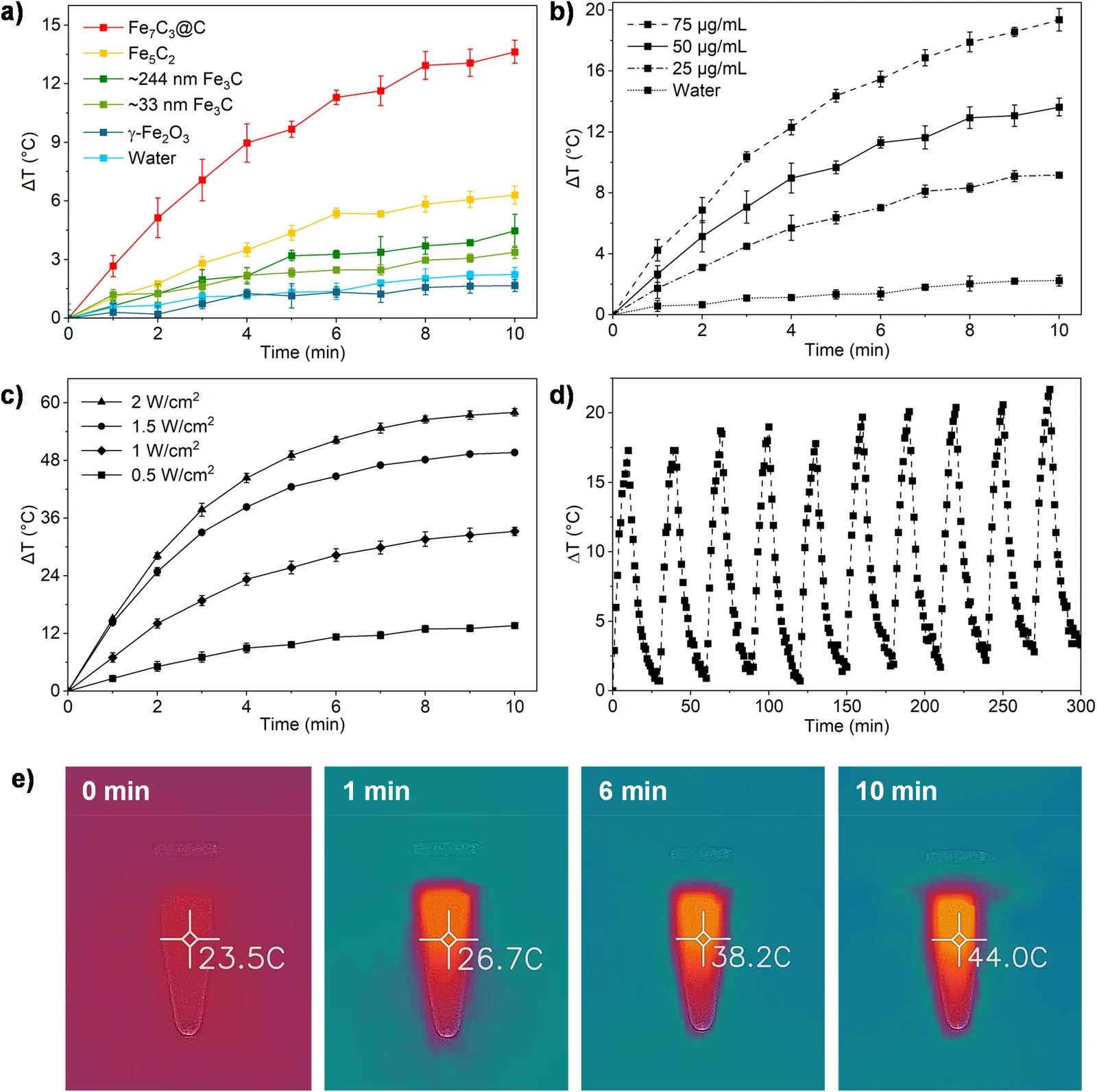
All the measurements in (a)–(c) were performed in triplicate. (a) Temperature increase of Fe_7_C_3_@C, Fe_5_C_2_ and Fe_3_C NPs under 808 nm irradiation (50 μg mL^−1^, 0.5 W cm^−2^). (b) Temperature increase of Fe_7_C_3_@C NPs at different concentrations under 808 nm laser irradiation (0.5 W cm^−2^). (c) Temperature increase of Fe_7_C_3_@C NPs (50 μg mL^−1^) at different laser powers. (d) Temperature increase of Fe_7_C_3_@C NP solution (75 μg mL^−1^) under 808 nm laser irradiation (0.5 W cm^−2^) over 10 cycles. (e) Thermal camera pictures of Fe_7_C_3_@C NPs (75 μg mL^−1^) captured at 0, 1, 6, and 10 min at 0.5 W cm^−2^ (Δ*T* = 0, 3, 15, and 20 °C), respectively.

Furthermore, control experiments performed on γ-Fe_2_O_3_ NPs of comparable size, known to display limited heating efficiency,^48^ showed only a limited photothermal response (Δ*T* ≈ 1.7 °C), demonstrating that the oxide by-products contribute negligibly to the overall heating of the Fe_7_C_3_@C sample and that the intrinsic photothermal performance of Fe_7_C_3_@C could be even higher. Then, the Fe_7_C_3_@C NP concentration was varied from 25 to 75 μg mL^−1^, leading to a temperature increase from 9.2 to 19.4 °C after 10 min of laser irradiation (Fig. 4b and e). In Fig. 4c, a power-density-dependent increase in temperature was observed for Fe_7_C_3_@C NPs at 50 μg mL^−1^, reaching 58 °C at 2 W cm^−2^. Finally, the cyclability of the heat generation under laser irradiation was tested. Specifically, Fe_7_C_3_@C (75 μg mL^−1^) NP solution was irradiated (0.5 W cm^−2^) for 10 min and then cooled down to room temperature before being irradiated again. This procedure was repeated 10 times. Fig. 4d shows that the maximum temperature reached over 10 min is similar across cycles, demonstrating the stability of the NPs under laser irradiation and the maintenance of their photothermal properties. Moreover, TEM images and XRD diffractograms acquired after ten irradiation cycles (Fig. S11 and S24, SI) show that the NPs retain both their morphology and crystalline phase, further confirming their stability. Finally, to extend the comparison of Fe_7_C_3_@C NPs to metallic nanostructures, the PCE (*η*) was calculated using Roper's approach^49^ with the following equation:
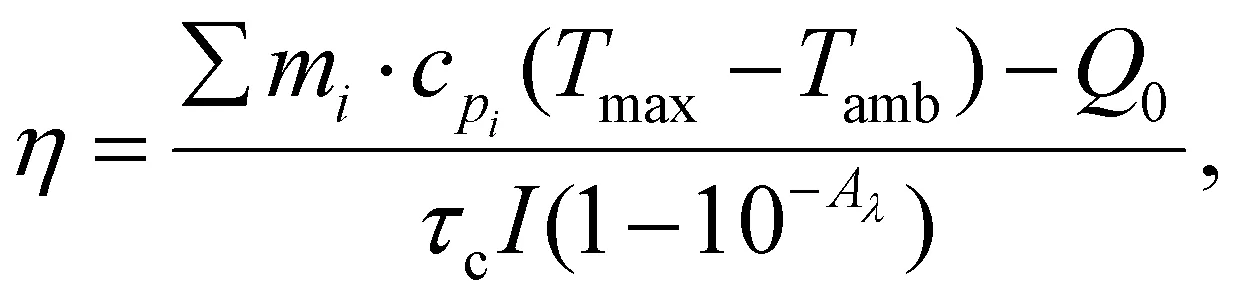
where ∑*m*_*i*_·*c*_*p*_*i*__ is the sum of the products of the mass by the heat capacity of each system component, *T*_max_ is the maximum temperature reached, *T*_amb_ is the ambient air temperature, *Q*_0_ is the heat transduced after irradiation of the solvent, which has been determined to be negligible for distilled water, *I* is the laser power (in W), and *A*_*λ*_ is the absorbance of the sample at the laser irradiation wavelength λ (Fig. S1, SI). *τ*_c_ is the cooling time constant, first introduced by Roper, and can be derived from *T*(*t*) − *T*_amb_ = (*T*_max_ − *T*_amb_)*e*^−*t*/*τ*_c_^ as the slope −1/*τ*_c_ in the linear fitting of 
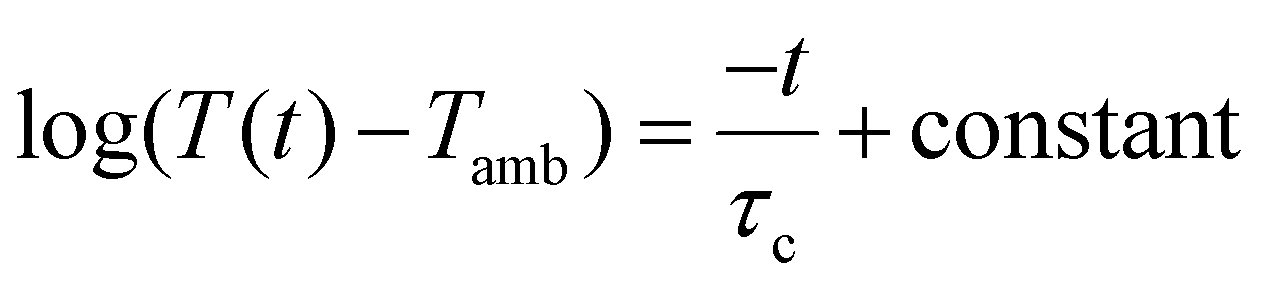
 (Fig. S2, SI).

A PCE of 84 ± 4% was obtained from three measurements (details of the calculations and all fitting parameters are provided in the SI), significantly higher than those of other iron carbide phases (12.4% for Fe_2_C and 36.8% for Fe_5_C_2_)^5,20^ and even superior to those of various metallic nanostructures (52% for Pd nanosheets, 48.5% for Co_3_O_4_ NPs, and 48.7% for a gallic acid–Ag NP hydrogel).^50^ Notably, this value also exceeds that of gold nanorods,^20^ with lengths above ∼40 nm, whose PCEs decrease with an increasing aspect ratio, although it remains inferior to those of shorter gold nanorods (∼95%) and gold NPs (∼97%).^51^ The PCE of Fe_7_C_3_ exceeds those of other magnetic NPs, such as PEGylated Fe_3_O_4_ NPs (15.9–16.9%),^52^ Mn^2+^-doped iron oxide NPs (26.9%),^53^ and bare Fe_3_O_4_ NPs (6.4%).^54^ It is also notably higher than those of several carbon-based materials, such as carbon spheres (54.2%),^55^ assembled carbon nanodots (52%),^56^ graphene oxide and graphene (58 and 67%, respectively).^57^

## 4. Conclusions

In this work, we demonstrated that Fe_7_C_3_ uniquely induces the *in situ* formation of conformal graphitic shells during a one-pot solvothermal synthesis under comparatively mild conditions. Comprehensive structural and spectroscopic analyses revealed that these NPs consist of a crystalline Fe_7_C_3_ core encapsulated in a few-layer graphitic carbon shell, with small maghemite NPs formed as by-products. Systematic variation of the reaction parameters showed that the Fe_7_C_3_ phase and a reducing atmosphere are essential for graphitisation. Indeed, replacing NH_3_ with Ar led exclusively to iron oxide formation, whereas Fe_5_C_2_ and Fe_3_C synthesised under analogous solvothermal conditions were coated only with an amorphous carbon shell. These results indicate that the primary role of NH_3_ is to act as a reducing agent. Under the synthesis conditions, NH_3_ dissociates into N_2_ and H_2_, with the latter maintaining a reducing atmosphere, avoiding the formation of oxidised species, while the iron in the Fe_7_C_3_ phase catalyses the formation of the graphitic layers. This structural difference directly translates into enhanced photothermal behaviour. Under irradiation at 808 nm, the Fe_7_C_3_@C NPs exhibited a much higher temperature increase than the Fe_5_C_2_ and Fe_3_C NPs at equal concentrations, while retaining similar heating performance after 10 irradiation cycles. The Fe_7_C_3_@C NPs exhibited a PCE of 84 ± 4%, exceeding those of other iron carbide phases and outperforming several metallic nanosystems. Overall, this work establishes Fe_7_C_3_ as a particularly promising iron carbide phase for photothermal applications and demonstrates that phase-dependent carbon ordering can be leveraged to tune the optical and thermal responses in iron carbide NPs.

## Author contributions

CP and LR: synthesis, investigation, structural characterisation, writing of the original manuscript, final review, and supporting information. EF and CMM: photothermal analyses, Raman characterisation, review and editing. JD: TEM imaging. CR and JK: supervision, review and editing. JMG: Mössbauer characterisation and analysis. BB: TEM, STEM and EELS characterisation. LM: conceptualisation, supervision, review, editing, and funding acquisition. DB: conceptualisation, project administration, supervision, writing original manuscript, review and editing, and funding acquisition.

## Conflicts of interest

The authors declare no conflicts of interest.

## Supplementary Material

NR-018-D6NR01713D-s001

## Data Availability

Supplementary information (SI): ^57^Fe Mössbauer spectrometry, TEM characterization, vis-NIR spectroscopy, PCE calculation, size distribution, SQUID characterization, and X-ray diffraction. See DOI: https://doi.org/10.1039/d6nr01713d. Other data supporting this article are available upon request.
