# Supplementary material for: One-pot graphitic encapsulation of Fe7C3 nanoparticles enabling enhanced photothermal performance
Source: Nanoscale. 2026 Aug 28;18(40):21978–90. doi: 10.1039/d6nr01713d (PMC13617640; doi:10.1039/d6nr01713d)
Supplement: Supplementary file 1 [file NR-018-D6NR01713D-s001.pdf]

Supplementary Information (SI) for Nanoscale.
This journal is © The Royal Society of Chemistry

Supporting information

One-pot graphitic encapsulation of Fe_7C_3 nanoparticles enabling enhanced photothermal performances

Chiara Puccinelli, † [a] *Lorenzo Riccio,* † [a] *Erica Frostegård,* [b] *Jana Dzíbelová,* [c] *Christian Rentenberger,* [c] *Jani Kotakoski,* [c] *Jean-Marc Greneche,* [d] *Belén Ballesteros,* [e] *Cécilia Ménard-Moyon,* [b,f] *Laura Maggini* [a] *and Davide Bonifazi* *[a]

† These authors contributed equally to this work.

* Corresponding author. davide.bonifazi@univie.ac.at

Table of contents

1. ^{57}Fe Mössbauer spectrometry
2. TEM characterization
3. Vis-NIR spectroscopy
4. PCE calculation
5. Size distribution
6. SQUID characterization
7. X-Ray Diffraction

1. Mössbauer spectrometry

Sample	T	IS	Γ	QS or 2ε	B_{hf}	RAA	RAA
	K	mm/s	mm/s	mm/s	T	%	%
		± 0.02	± 0.02	± 0.02	± 1	± 2	± 2
Fe_7C_3	300	0.40	0.70	0.87	-	68	68
		0.30	0.28	0.28	-	12	
		0.27	0.28	0.66	-	12	32
		0.23	0.28	1.02	-	8	
	77	0.51	1.10	1.12		15	75
		<0.49>	-	<-0.00>	<43.4>	60	
		0.38	0.44	-0.00	24.2	8	
		0.35	0.44	0.13	19.7	10	25
		0.38	0.44	0.11	15.9	7	

Table S1: Refined values of hyperfine parameters estimated from Mössbauer spectra obtained at 300 and 77 K on Fe_7C_3 sample: IS isomer shift; Γ linewidth at half-heigth; QS quadrupole splitting; 2ε quadrupole shift; B_{hf} hyperfine field; RAA relative absorption area.

Sample	T	IS	Γ	QS or 2ε	B_{hf}	RAA	RAA
	K	mm/s	mm/s	mm/s	T	%	%
		± 0.02	± 0.02	± 0.02	± 1	± 2	± 2
Fe_5C_2	300	0.40	1.00	1.21	-	34	34
		0.26	0.38	0.71	-	9	
		0.27	0.40	0.01	21.5	26	68
		0.17	0.32	0.08	19.8	16	
		0.22	0.38	0.01	17.8	15	
	77	0.51	1.20	1.25		6	34
		<0.48>	-	<-0.00>	<25.1>	28	
		0.35	0.41	0.08	25.0	32	
		0.32	0.55	0.04	22.3	29	66
		0.31	0.50	0.01	12.5	5	

Table S2: Refined values of hyperfine parameters estimated from Mössbauer spectra obtained at 300 and 77 K on Fe_5C_2 sample: IS isomer shift; Γ linewidth at half-heigth; QS quadrupole splitting; 2ε quadrupole shift; B_{hf} hyperfine field; RAA relative absorption area.

Sample	T (K)	IS (mm/s ± 0.02)	Γ (mm/s ± 0.02)	QS or 2ε (mm/s ± 0.02)	B_{hf} (T ± 1)	RAA (% ± 2)	RAA (% ± 2)
Fe_3C	300	0.34	0.90	1.10	-	8	8
		0.22	0.36	0.09	21.3	38	
		0.20	0.41	0.01	19.7	17	92
		0.21	0.43	0.07	17.9	22	
		0.19	0.31	0.16	11.0	15	11
	77	0.45	0.64	-0.11	52.3	11	11
		0.34	0.35	0.07	25.0	37	
		0.33	0.27	0.06	23.2	10	89
		0.37	0.66	0.11	21.1	32	
		0.36	0.36	0.15	13.0	10	

Table S3: Refined values of hyperfine parameters estimated from Mössbauer spectra obtained at 300 and 77 K on Fe_3C sample: IS isomer shift; Γ linewidth at half-heigth; QS quadrupole splitting; 2ε quadrupole shift; B_{hf} hyperfine field; RAA relative absorption area.

2. Vis-NIR spectroscopy

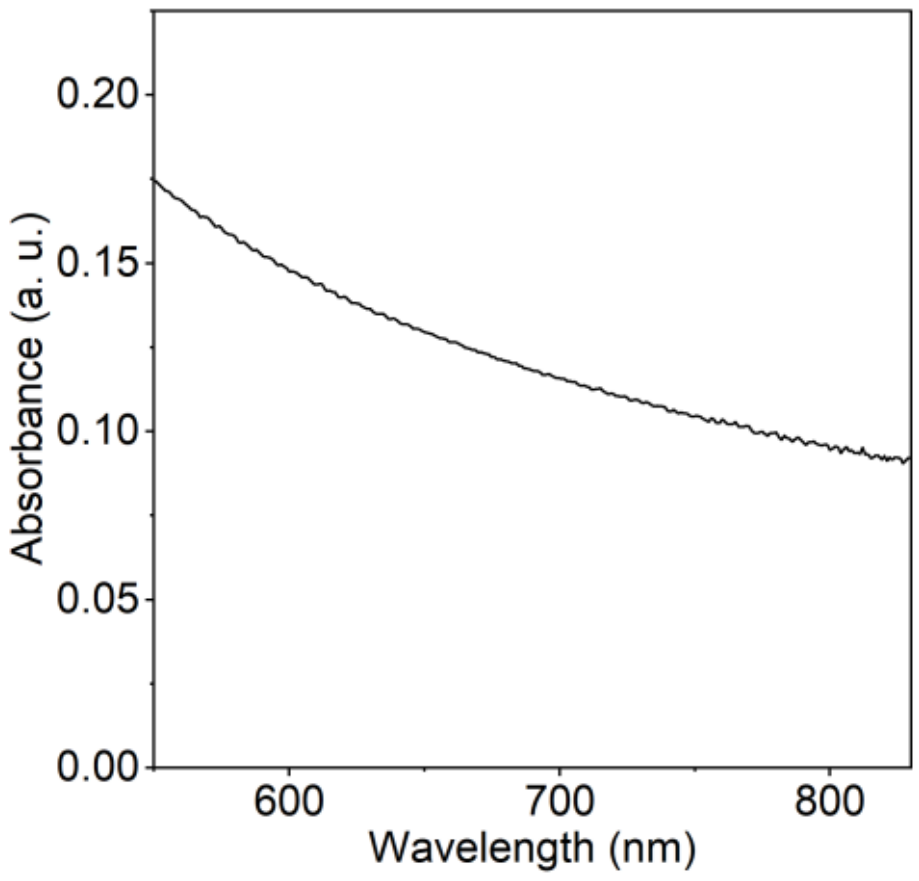

Figure S1 Absorbance spectrum of Fe_7C_3@C NPs in H_2O, concentration 75 μg/mL.

3. PCE calculation

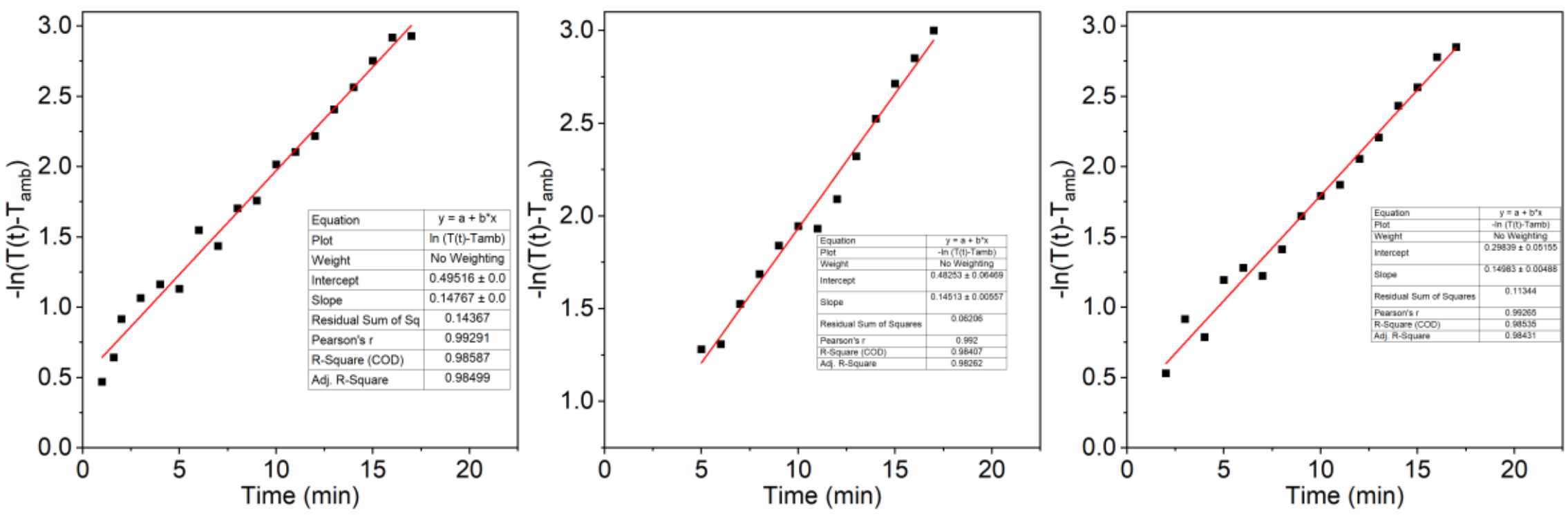

Figure S2 Linear fitting of the cooling rate of Fe_7C_3@C NPs in H_2O, concentration 75 μg/mL.

$$\eta = \frac{\sum m_i \cdot c_{p_i}(T_{max} - T_{amb}) - Q_0}{\tau_c I(1 - 10^{-A_y})}$$

$\sum m_i \cdot c_{p_i} = m_{Fe7C3@C} \cdot c_{p_{Fe7C3@C}} + m_{H2O} \cdot c_{p_{H2O}}$ = 75· 10^{-6} g (From Figure 4d) · 0.46 J/(g·K) + 0.5 g · 4.18 J/(g·K)

T_{max}-T_{amb}= 17.3 °C, 17.5°C, 15.7 °C (From Figure 4d)

Q_0= 0 (negligible for distilled water)

I = 300 mW (Material and Methods)

A_λ= 0.10022 (From Figure S1)

1/τ_c = 0.14767, 0.14513, 0.14983 (From Figure S2)

η=84 ± 4%

4. TEM characterization

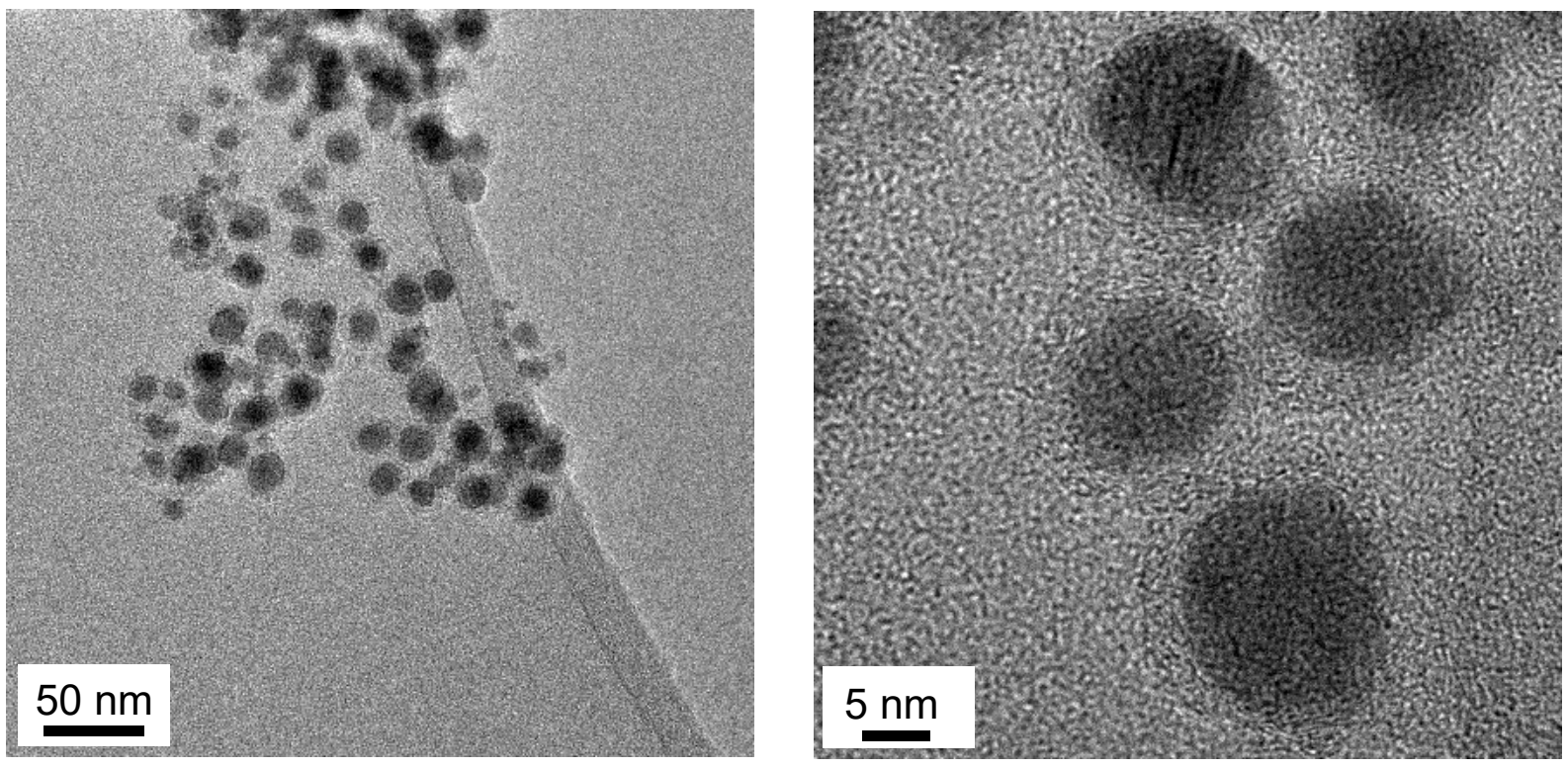

Figure S3 TEM images of Fe_7C_3@C NPs.

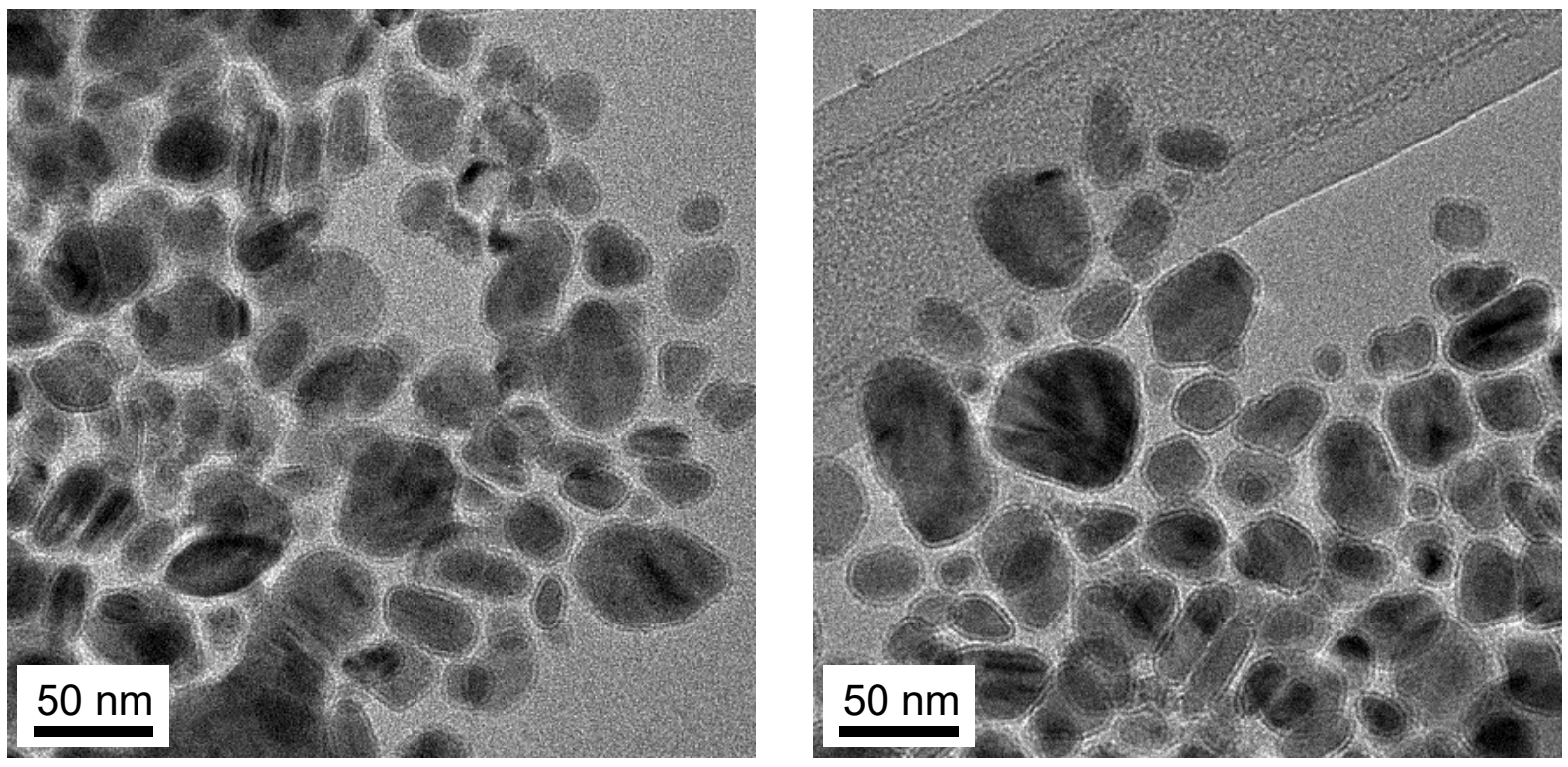

Figure S4 TEM images of Fe_5C_2 NPs.

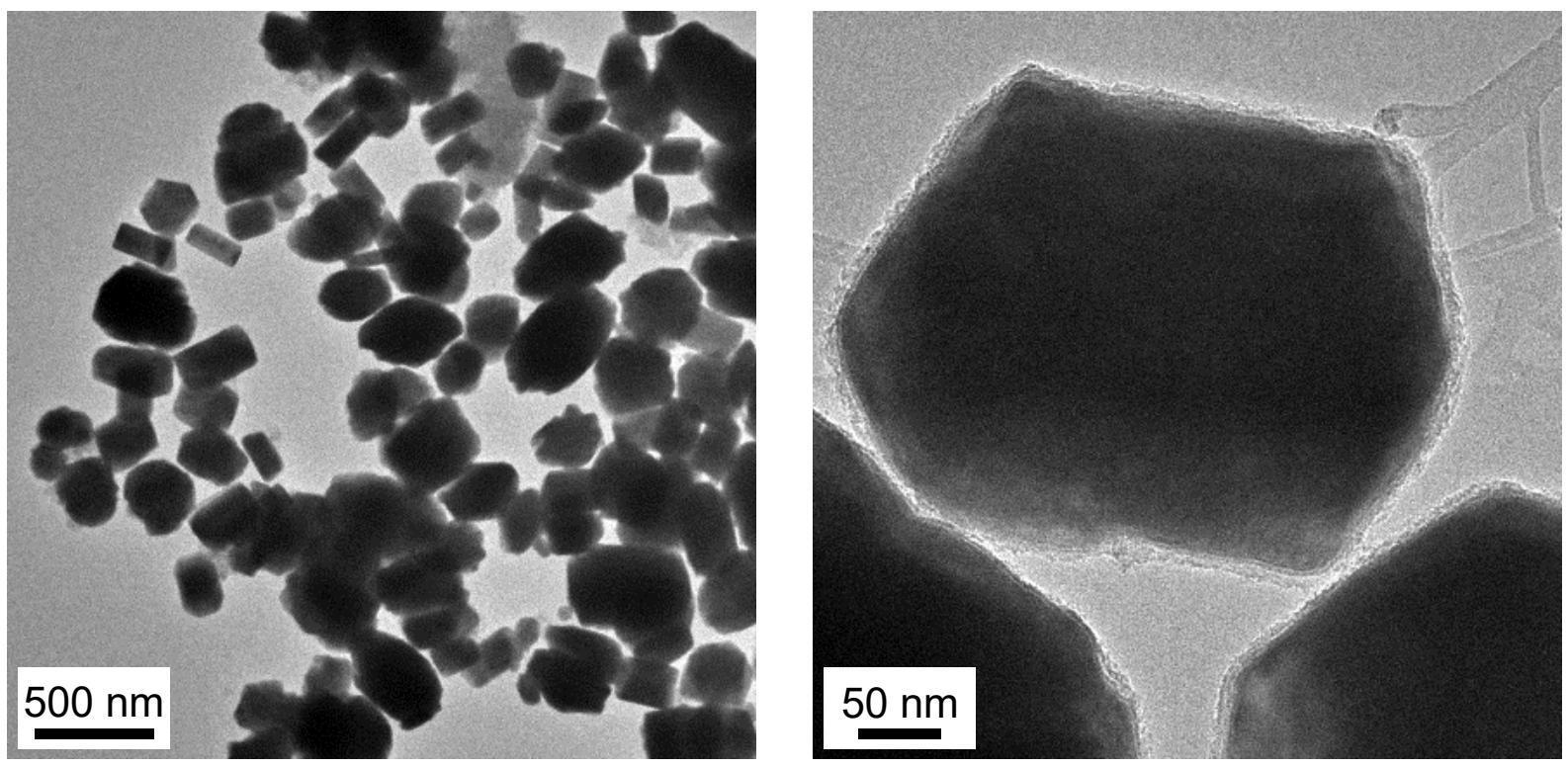

Figure S5 TEM images of one-pot synthetized Fe_3C NPs.

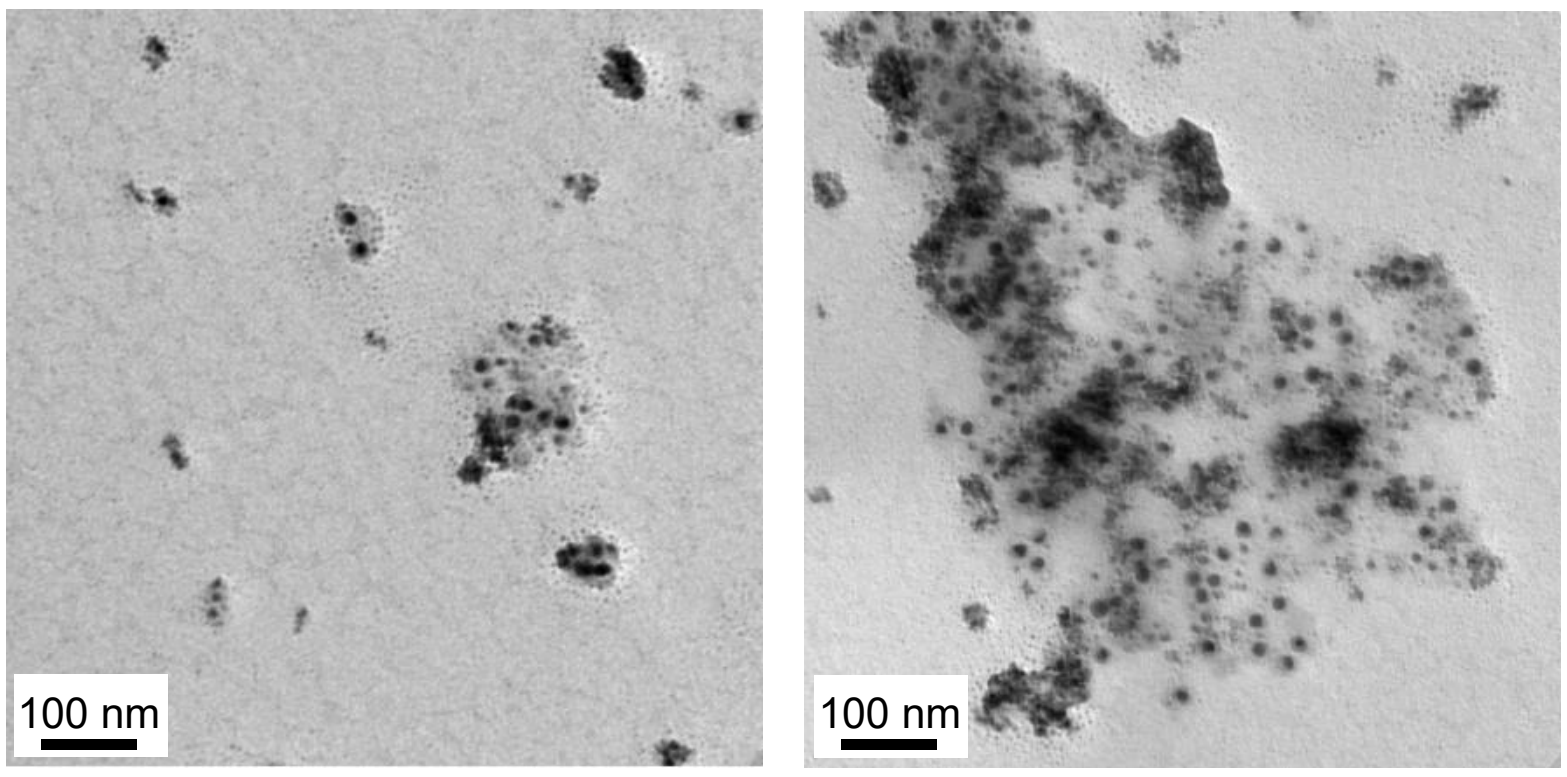

Figure S6 TEM images of DSPE-PEG(2000)-NH_2 coated Fe_7C_3@C NPs.

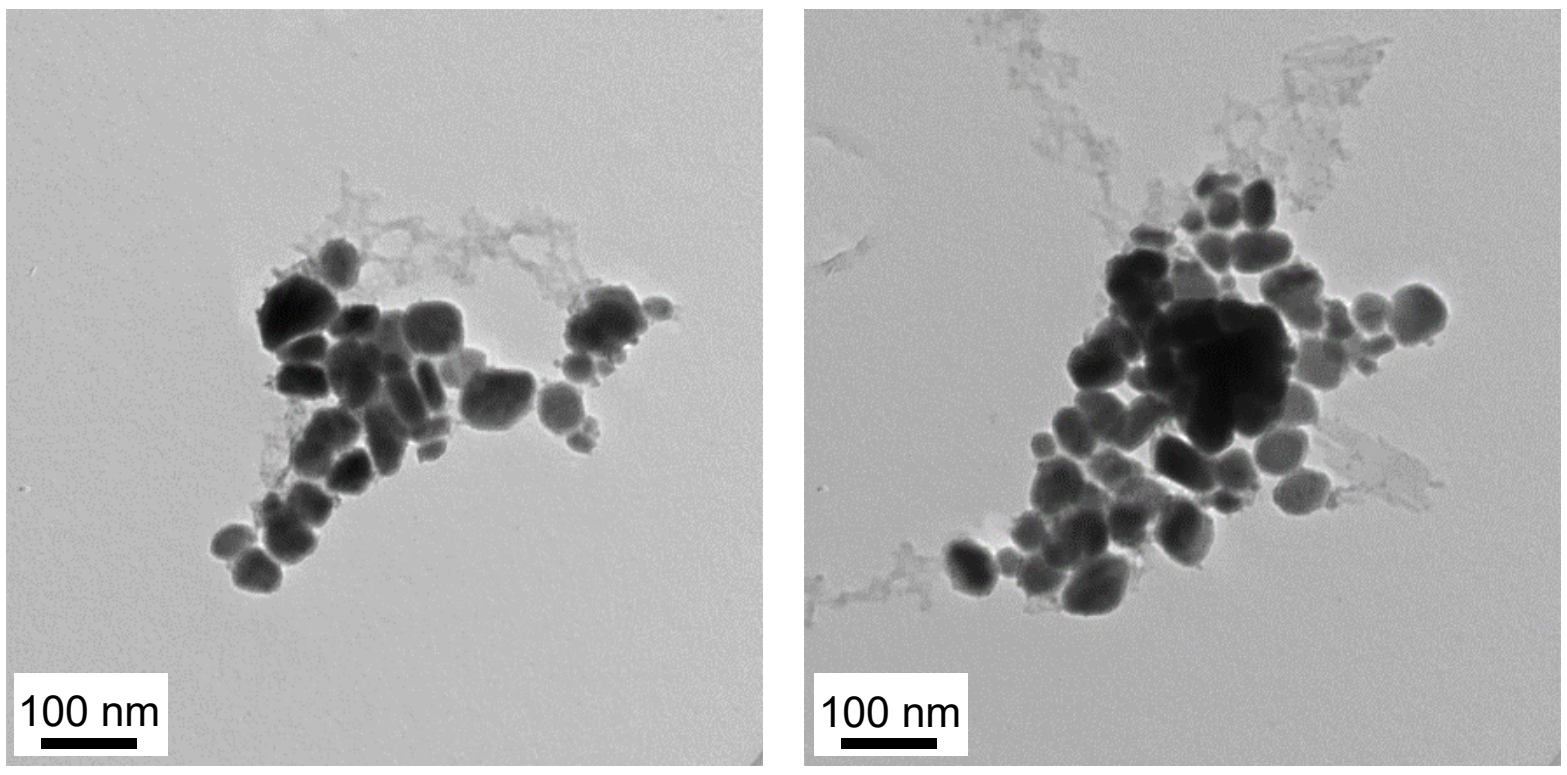

Figure S7 TEM images of DSPE-PEG(2000)-NH_2 coated Fe_5C_2 NPs.

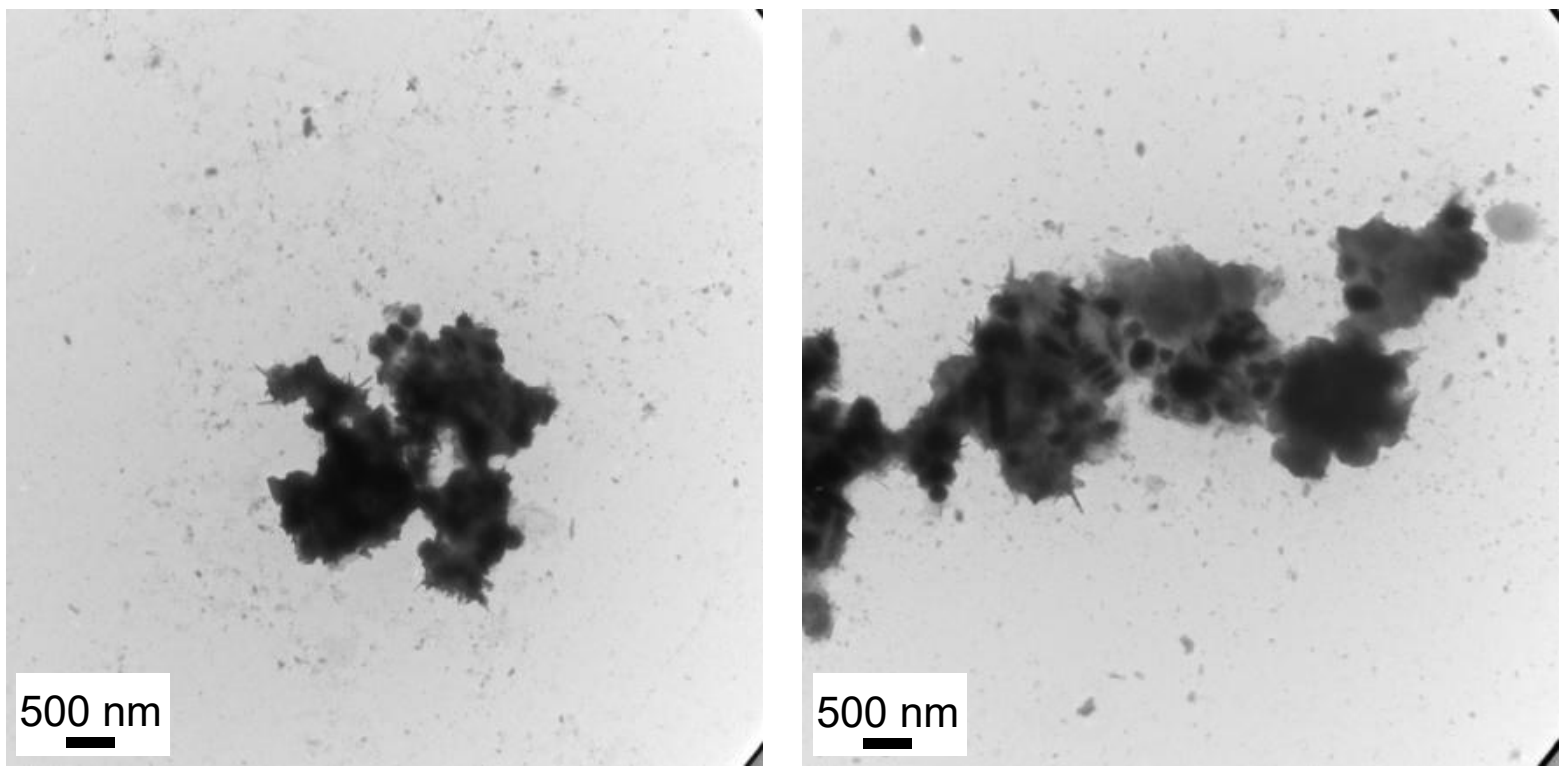

Figure S8 TEM images of DSPE-PEG(2000)-NH_2 coated Fe_3C NPs.

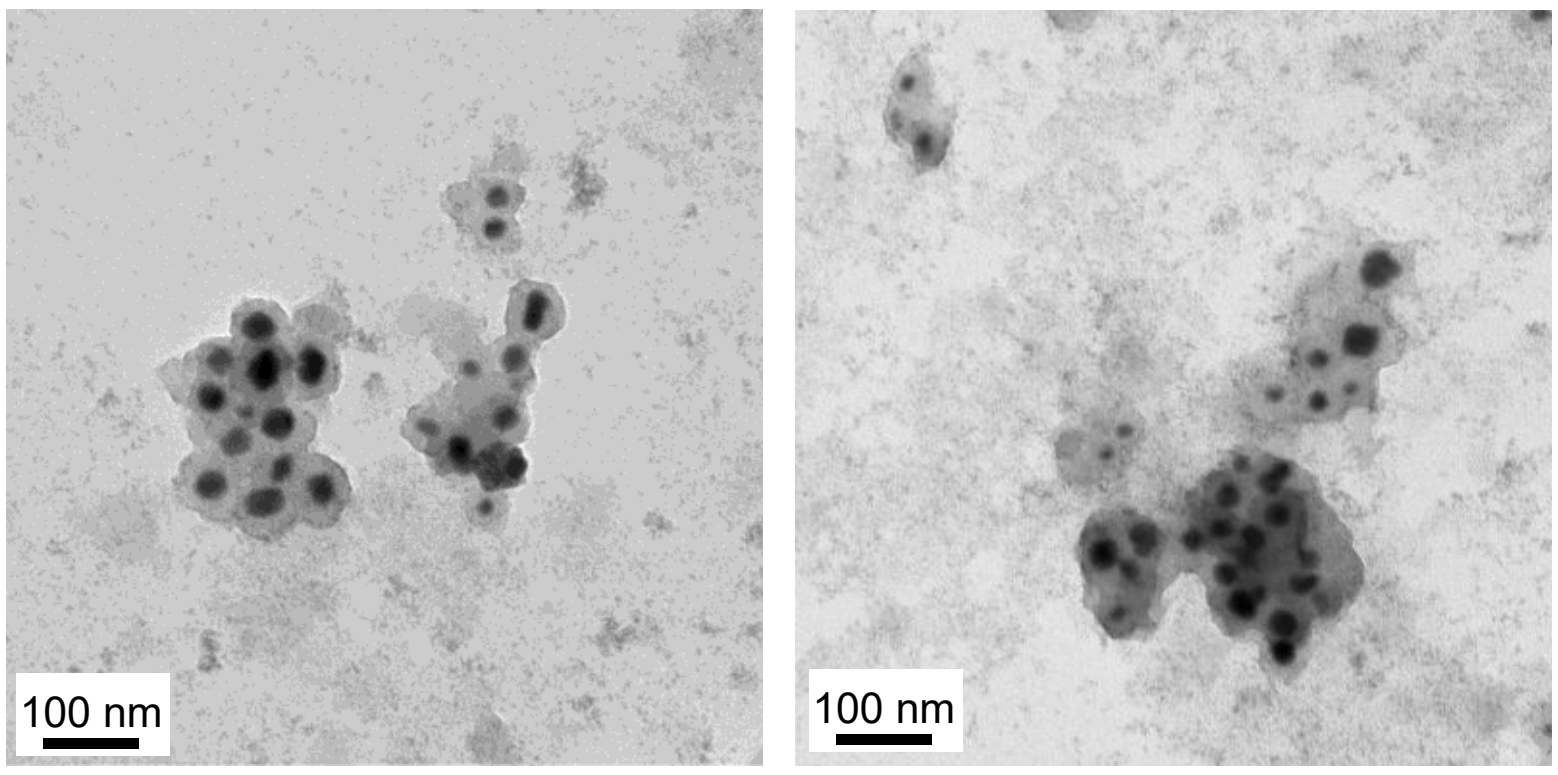

Figure S9 TEM images of DSPE-PEG(2000)-NH_2 coated two-step synthetized Fe_3C NPs.

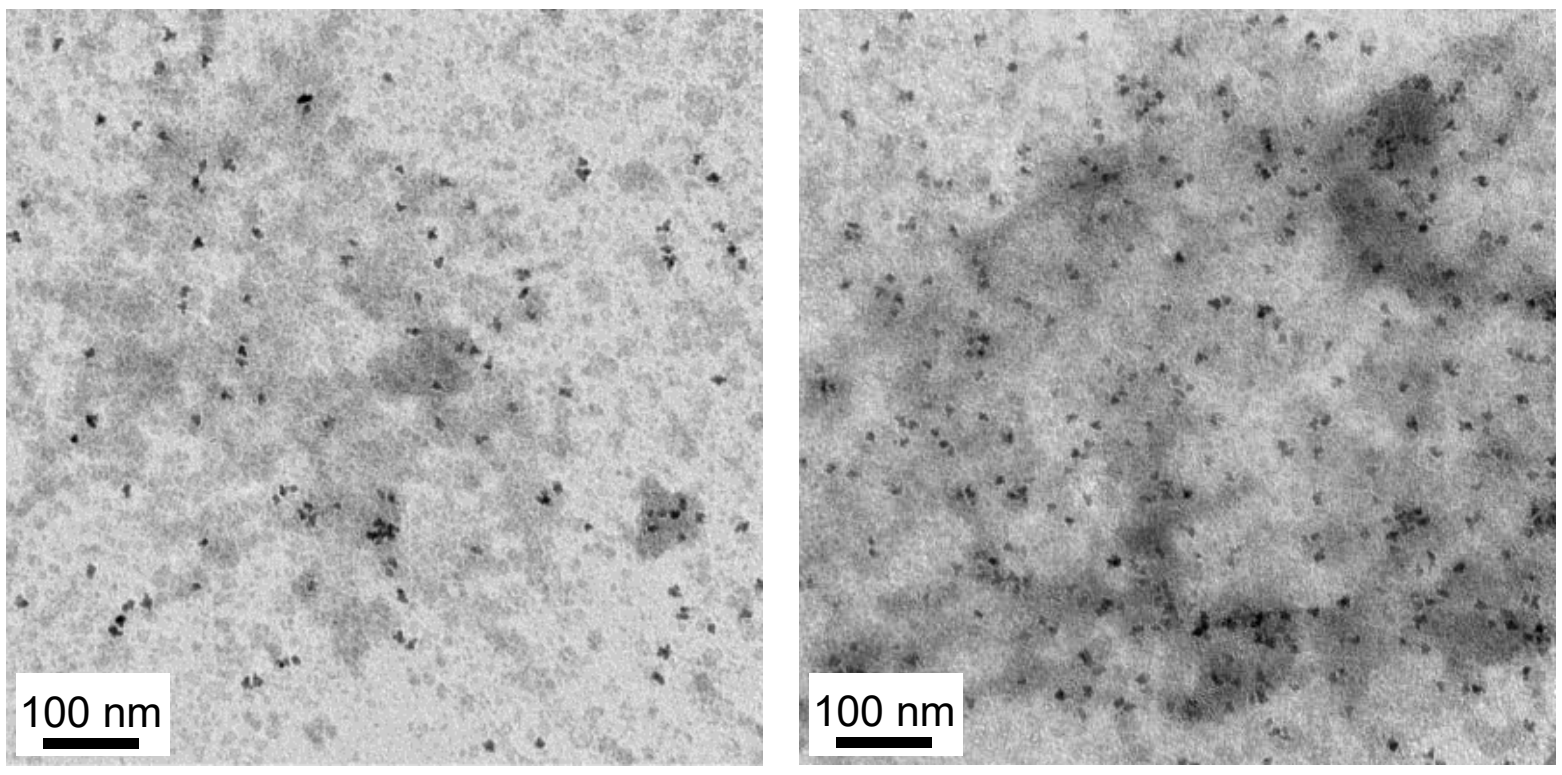

Figure S10 TEM images DSPE-PEG(2000)-NH_2 coated γ-Fe_2O_3 NPs.

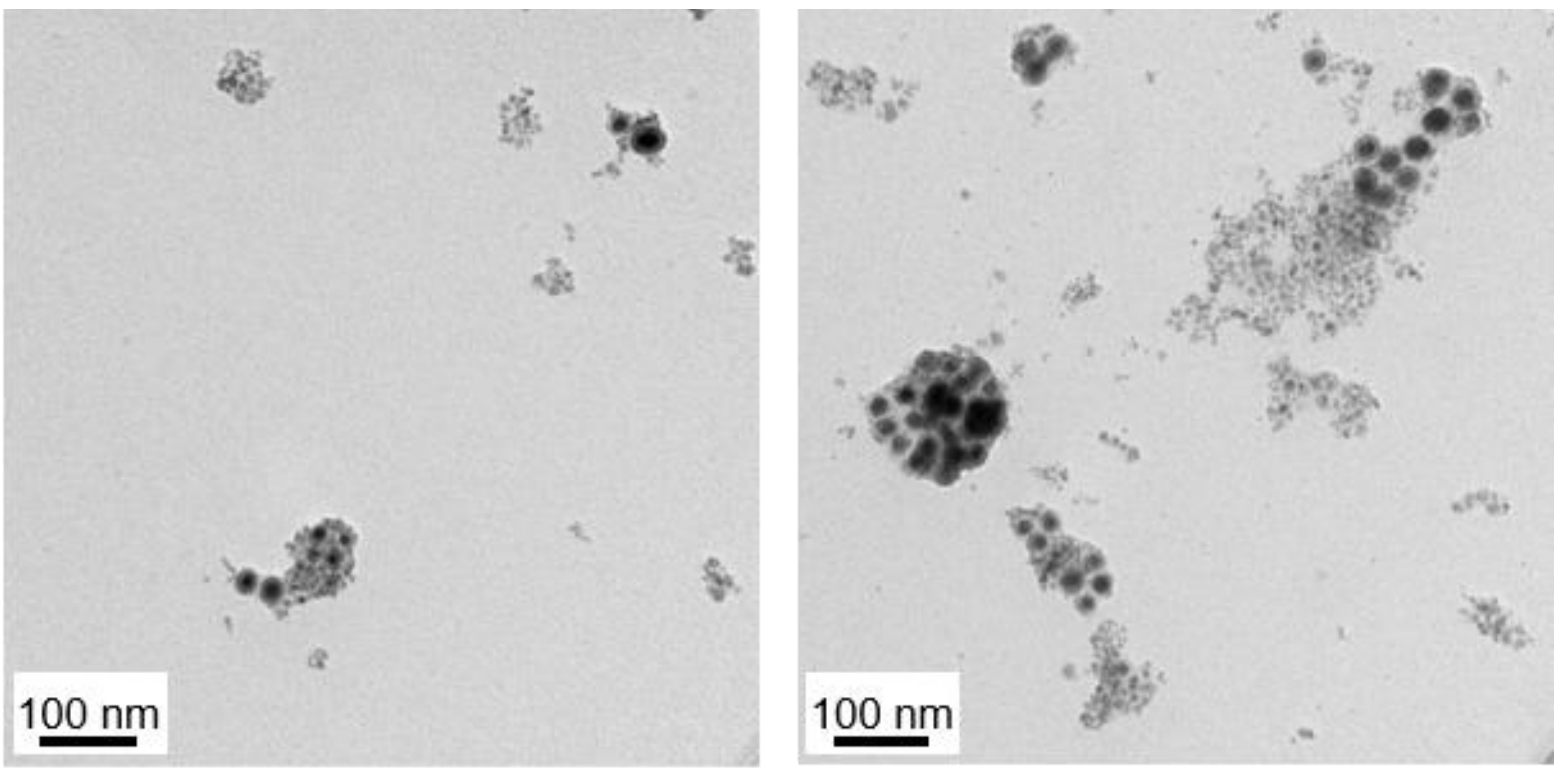

Figure S11 TEM images DSPE-PEG(2000)-NH_2 coated Fe_7C_3@C NPs after 10 cycles of irradiation.

5. Size distribution

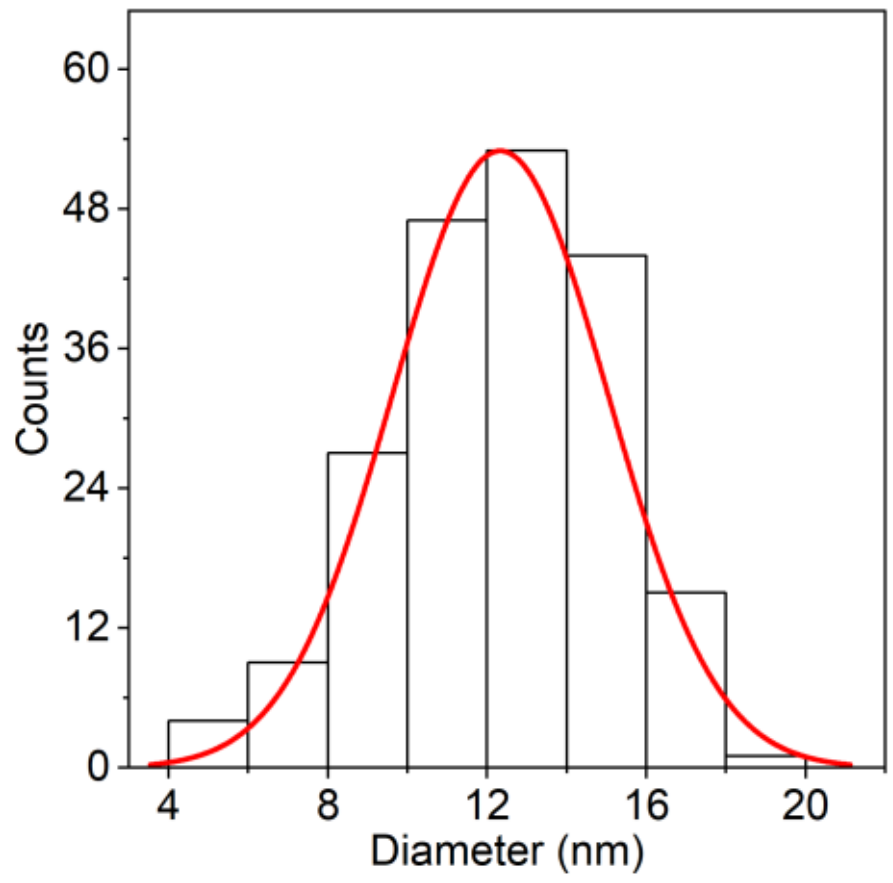

Figure S12 Size distribution of Fe_7C_3@C NPs (core) estimated from a set of 200 NPs.

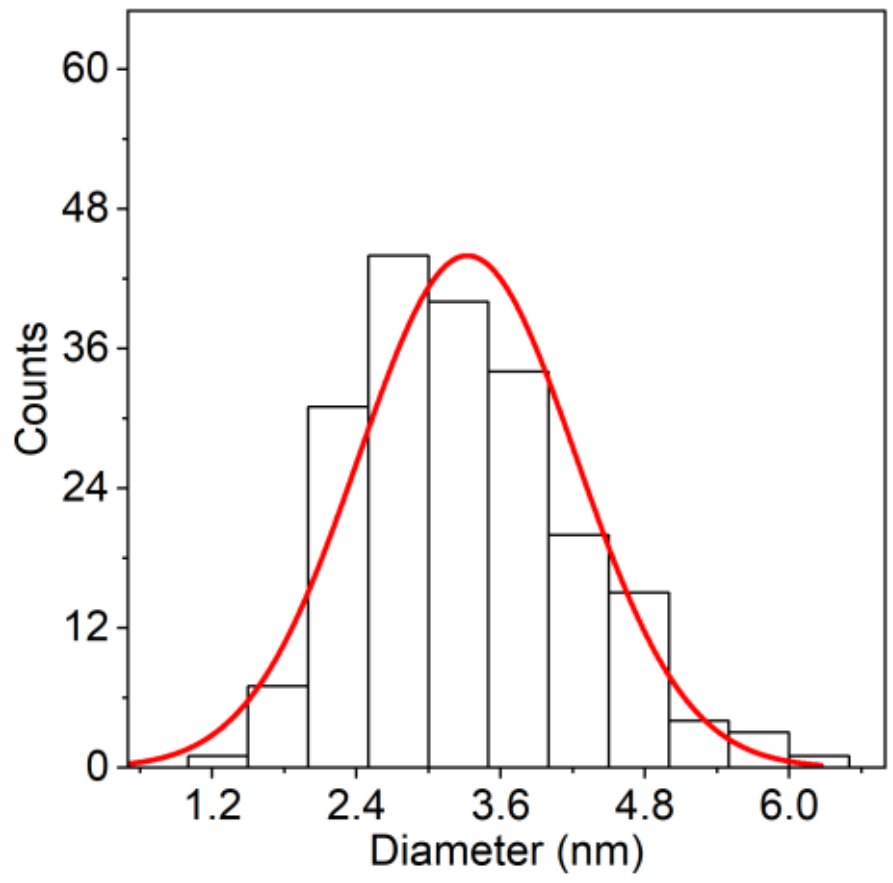

Figure S13 Size distribution of Fe_7C_3@C NPs (graphitic shell) estimated from a set of 200 NPs.

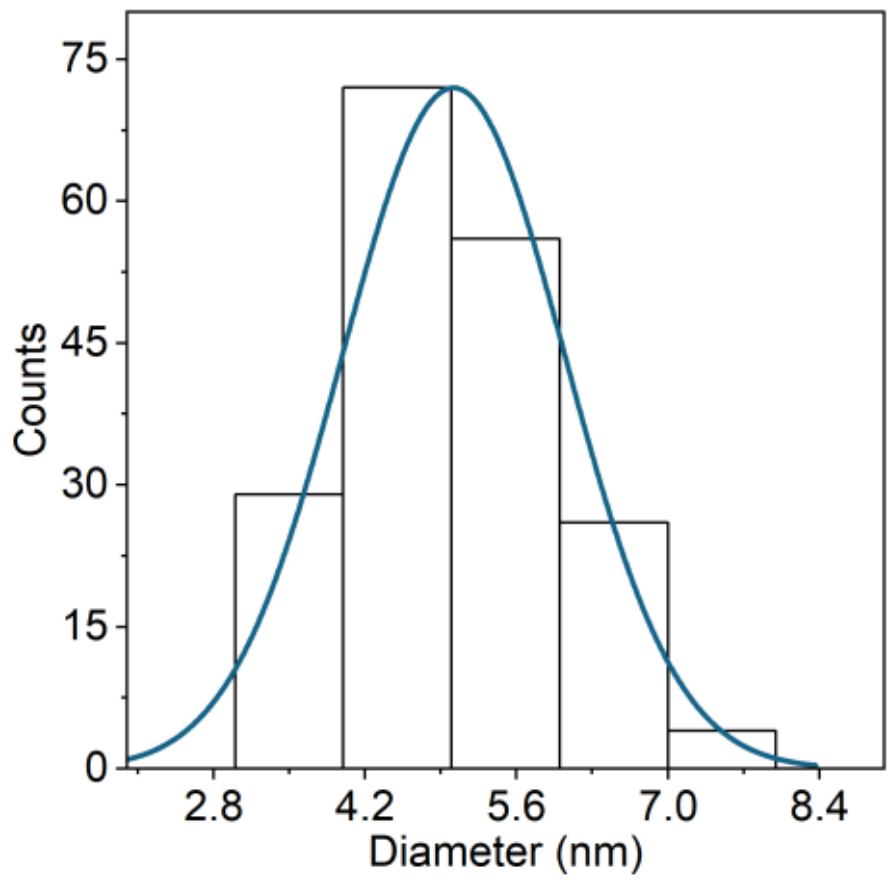

Figure S14 Size distribution of Fe_2O_3 NPs (in Fe_7C_3@C sample) estimated from a set of 200 NPs.

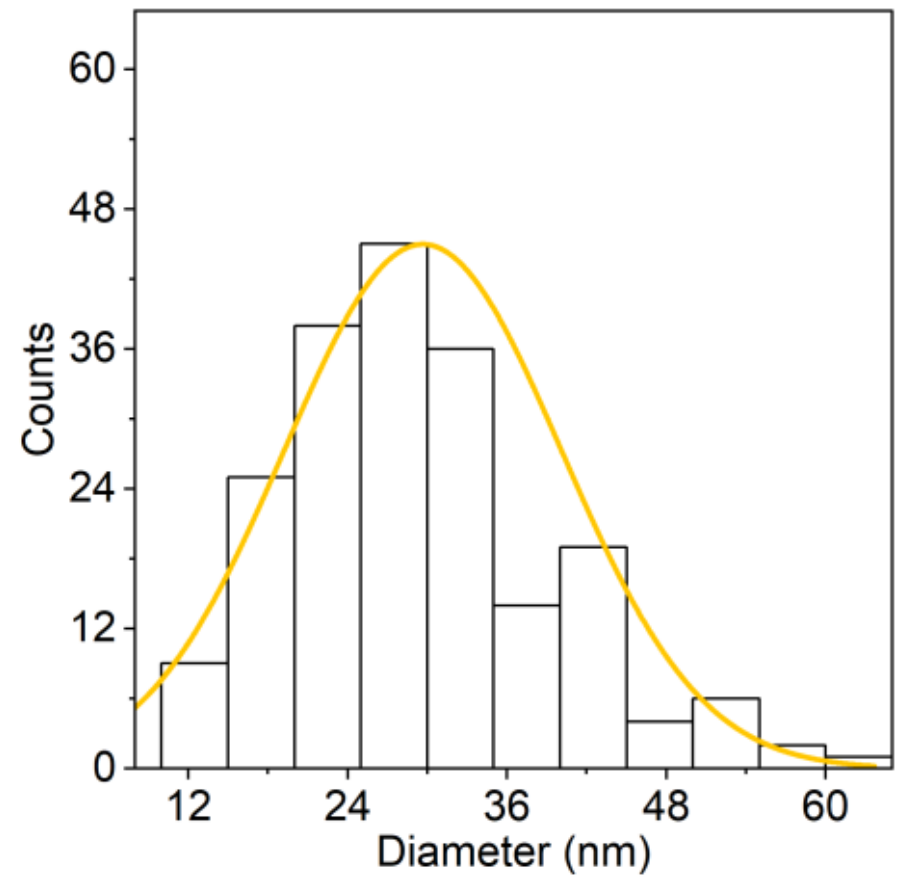

Figure S15 Size distribution of Fe_5C_2 estimated from a set of 200 NPs.

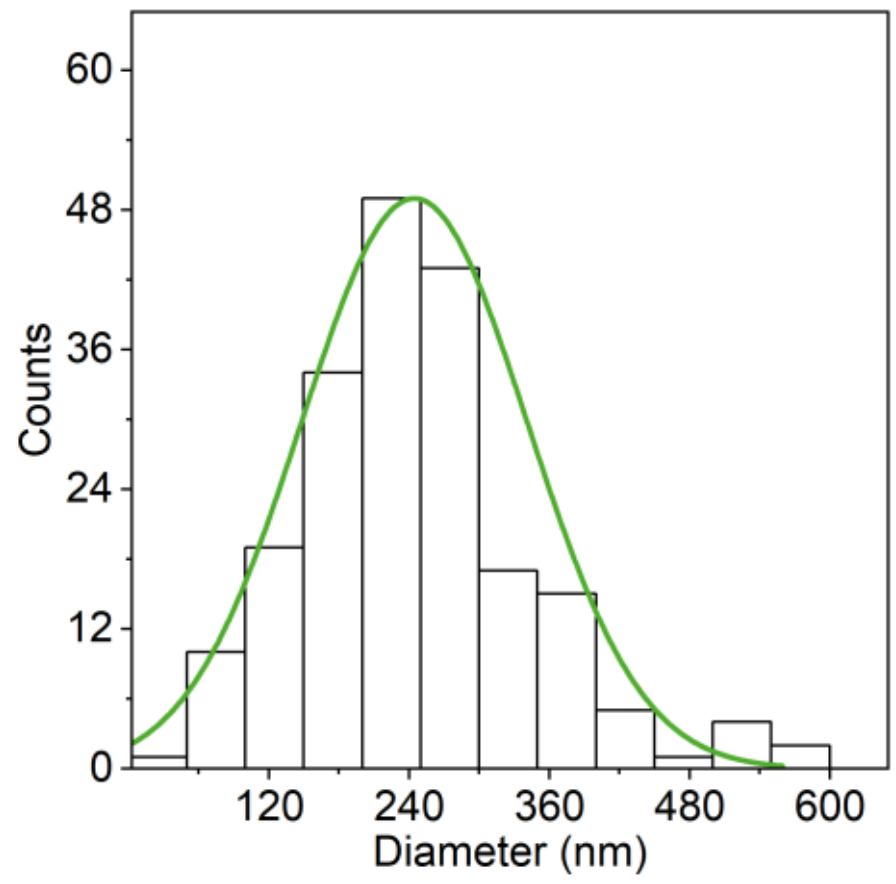

Figure S16 Size distribution of one-pot synthetized Fe_3C estimated from a set of 200 NPs.

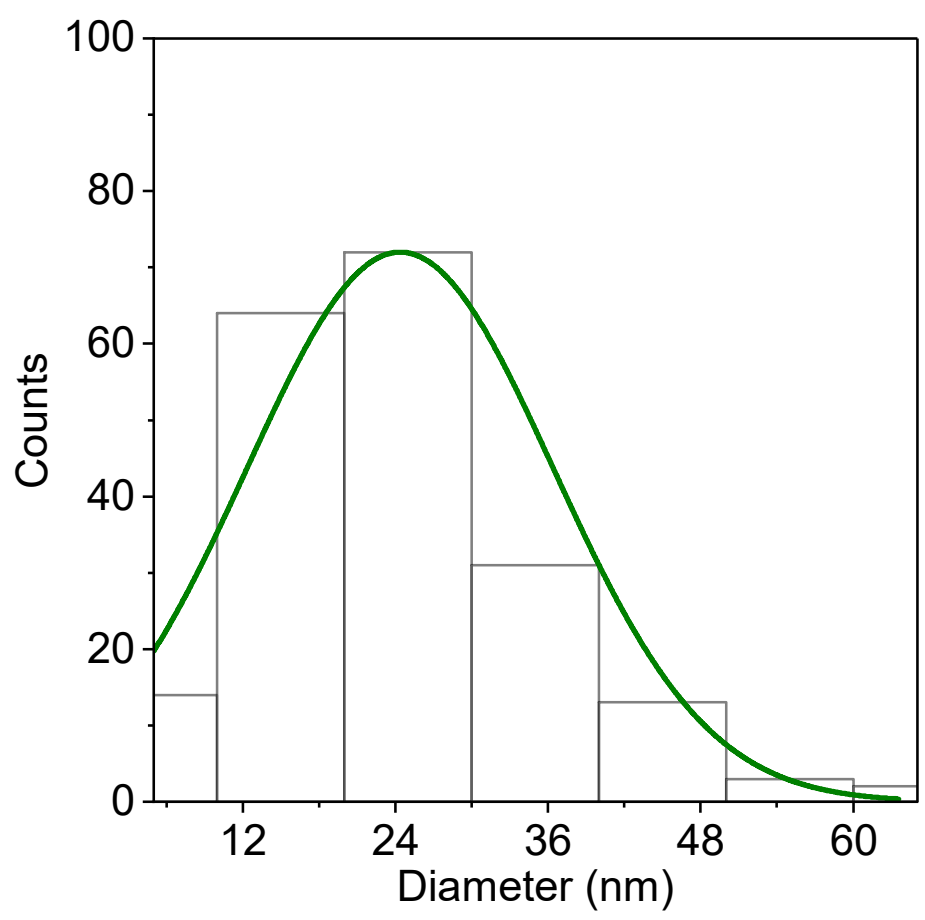

Figure S17 Size distribution of two-step synthetized Fe_3C estimated from a set of 200 NPs.

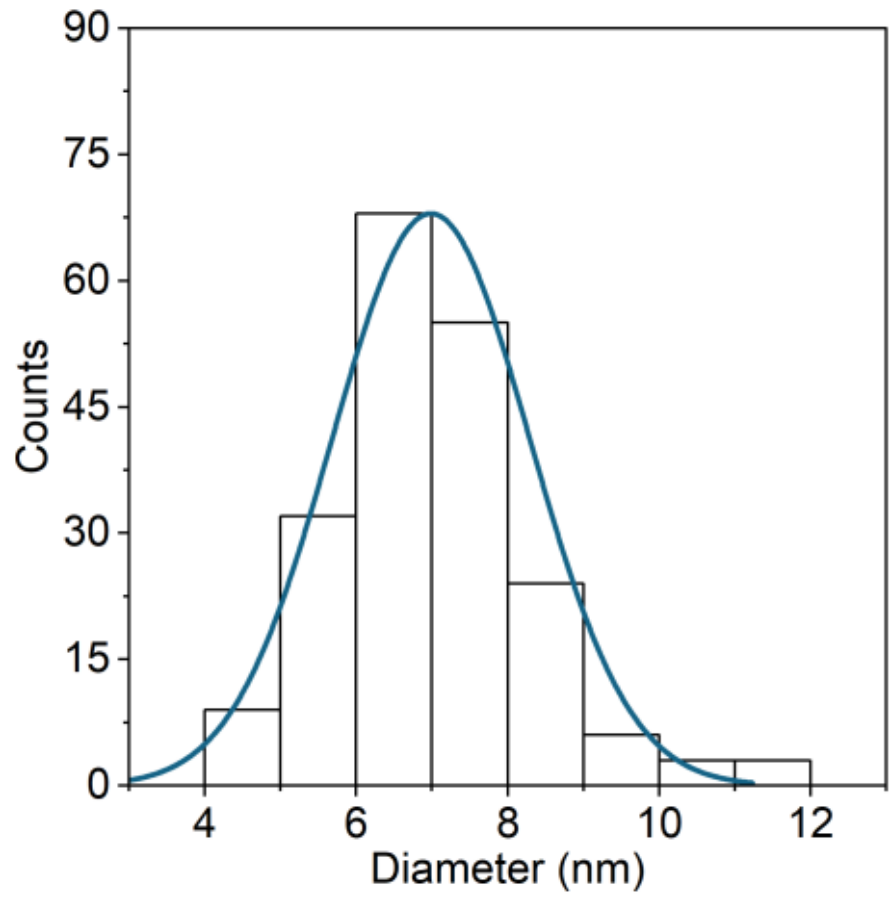

Figure S18 Size distribution of γ-Fe_2O_3 estimated from a set of 200 NPs.

6. SQUID characterization

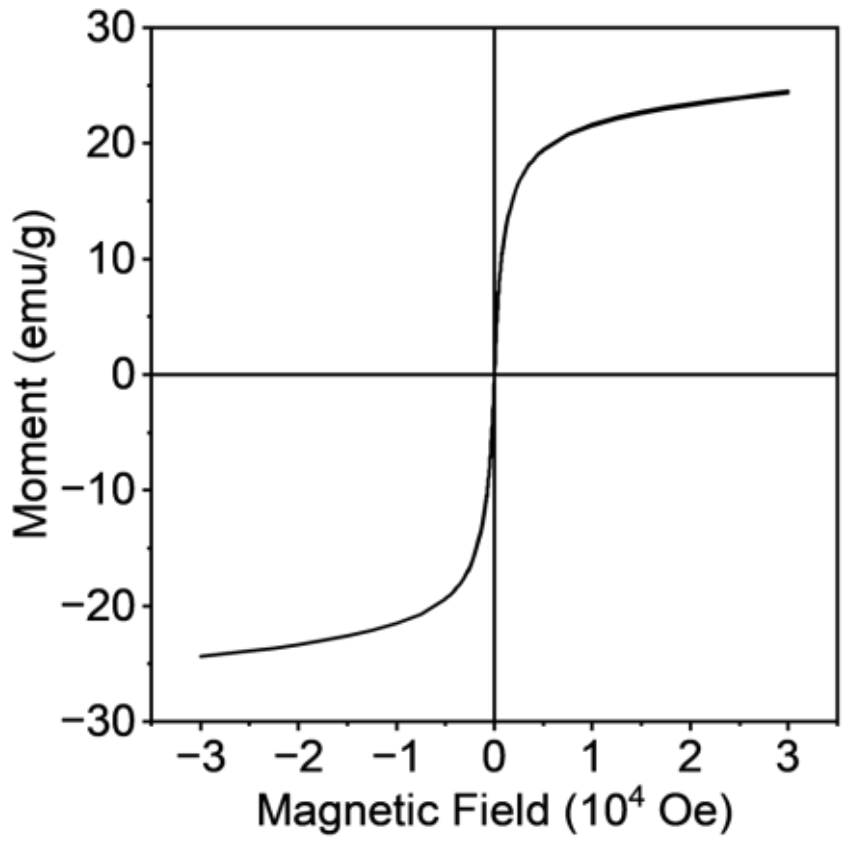

Figure S19 Superconducting quantum interference device measurements of Fe_7C_3@C NPs at room temperature.

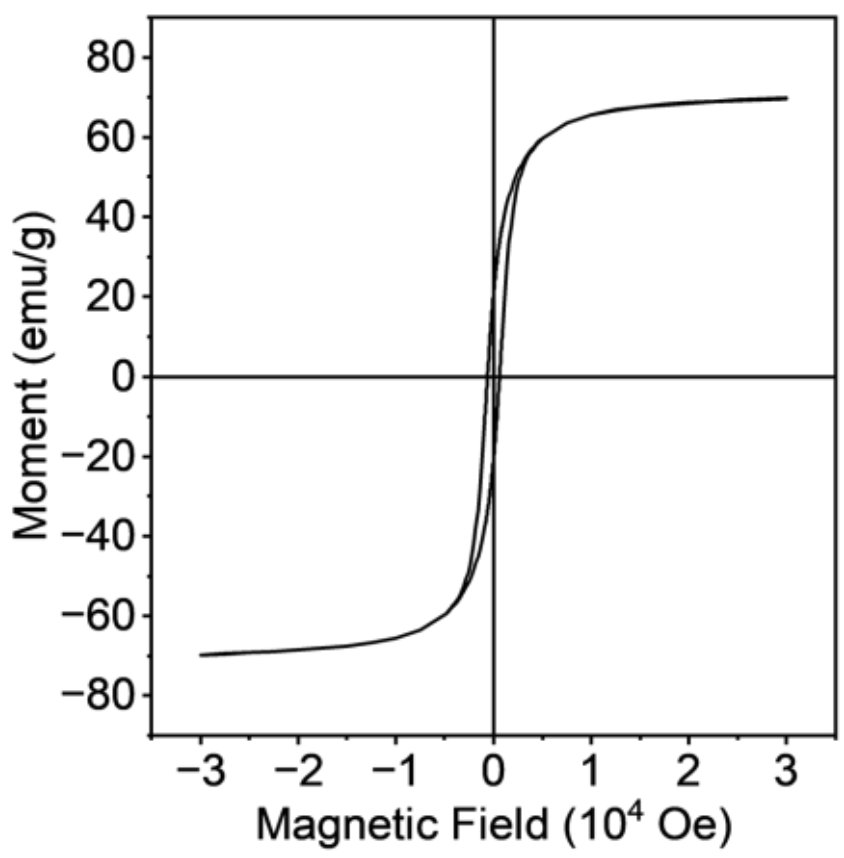

Figure S20 Superconducting quantum interference device measurements of Fe_5C_2 NPs at room temperature.

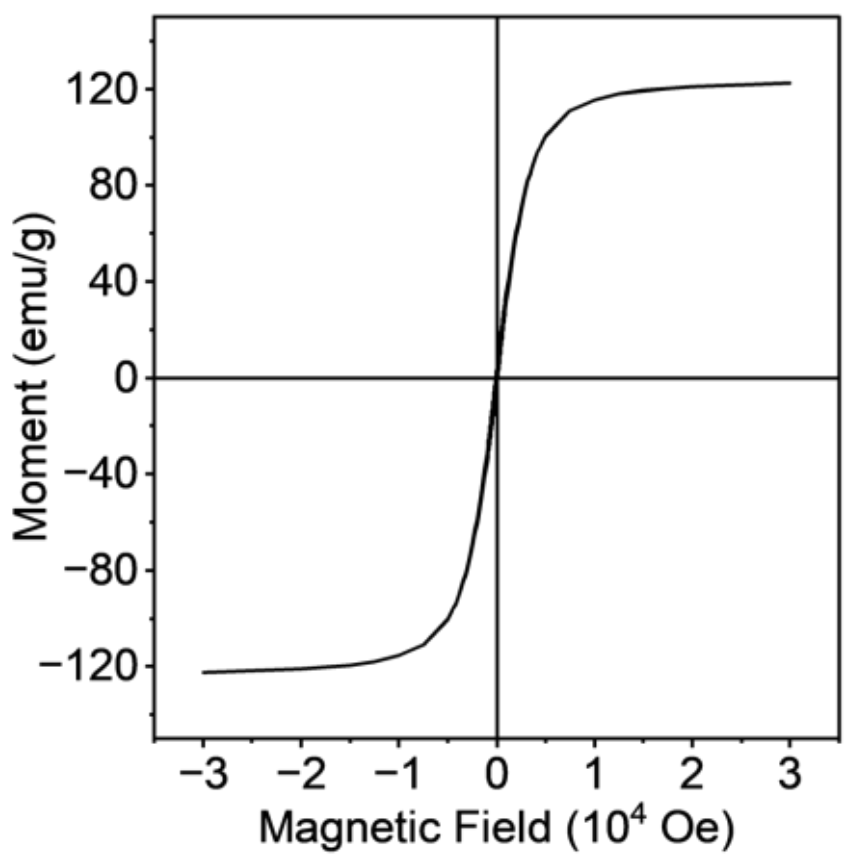

Figure S21 Superconducting quantum interference device measurements of Fe_3C NPs at room temperature.

7. X-Ray Diffraction

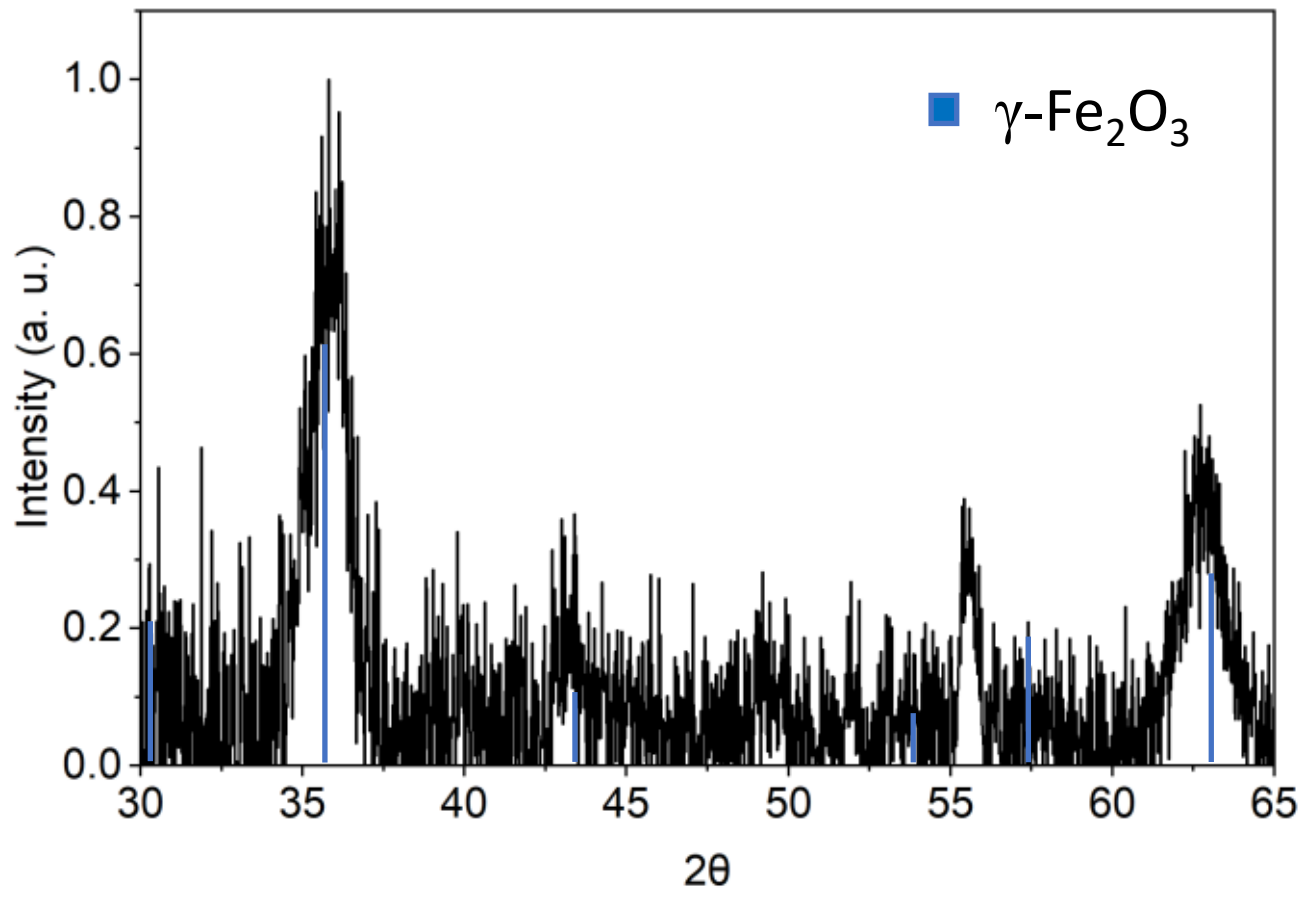

Figure S22. XRD diffractogram of γ-Fe_2O_3 NPs.

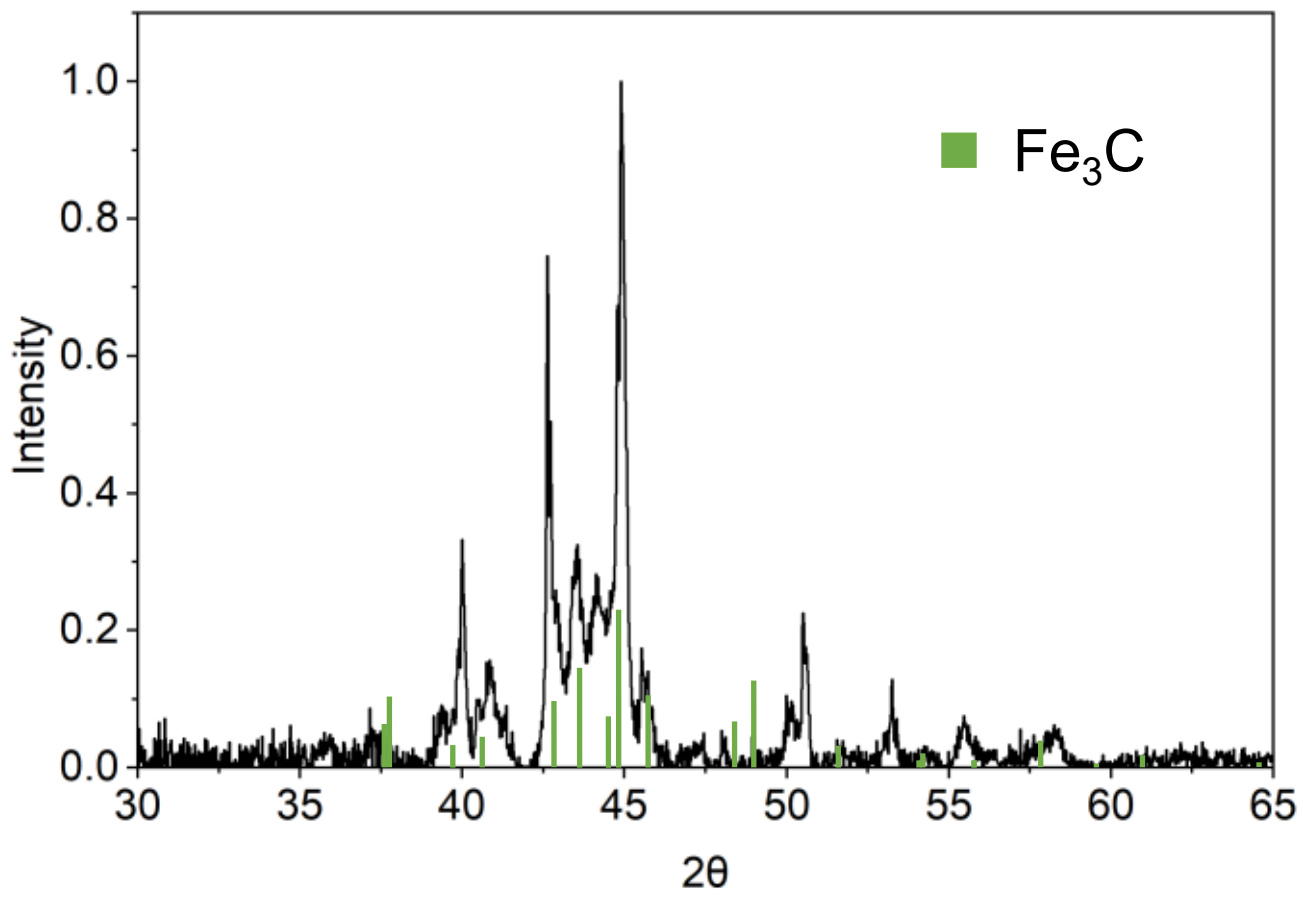

Figure S23. XRD diffractogram of γ-Fe_3C NPs.

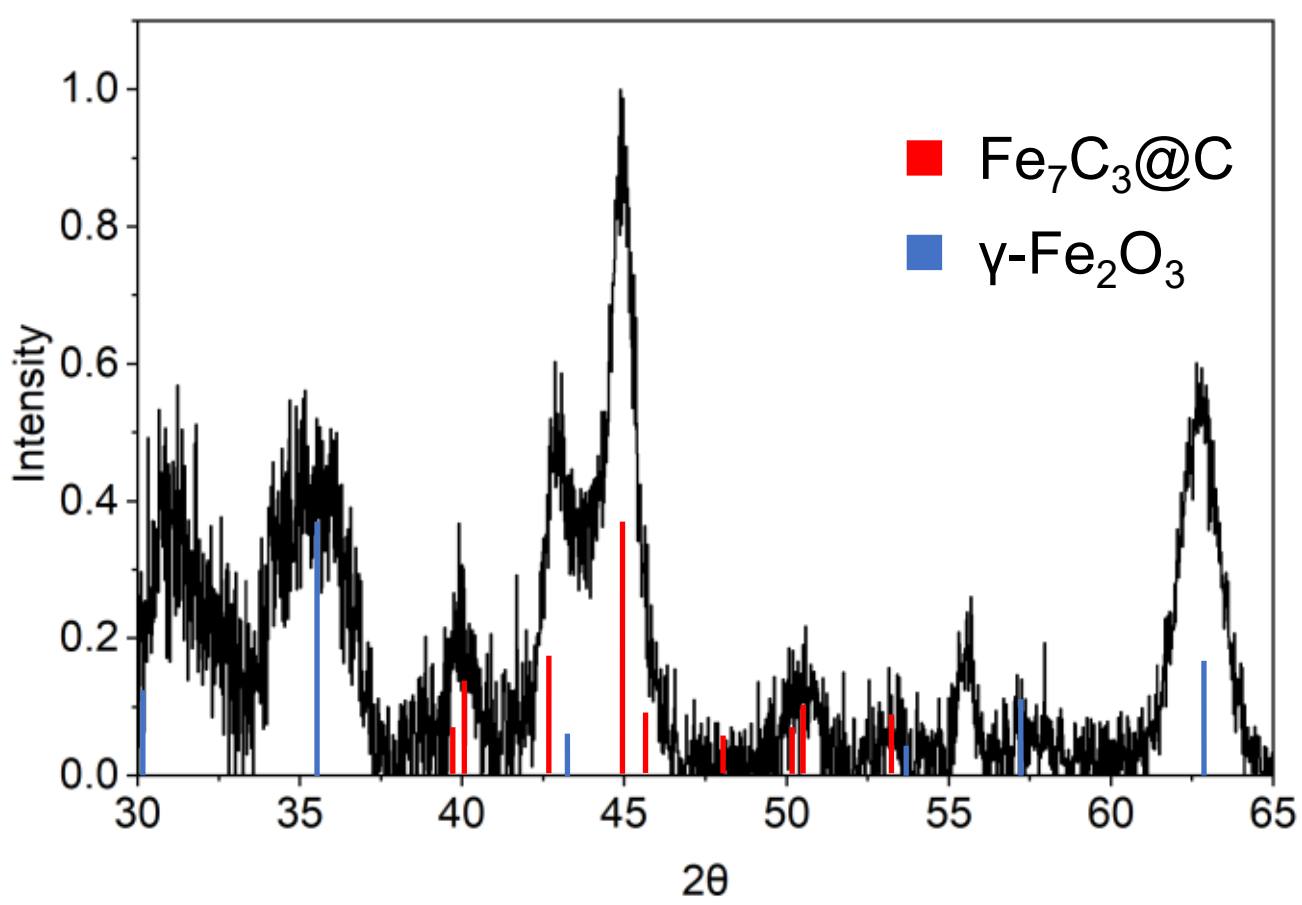

Figure S24. XRD diffractogram of DSPE-PEG(2000)-NH_2 coated Fe_7C_3@C NPs after 10 cycles of irradiation.
